# Protocol for isotype-resolved 3D imaging and quantification of bone marrow plasma cells in murine femurs

**DOI:** 10.1016/j.xpro.2026.104775

**Published:** 2026-08-10

**Authors:** Abdouramane Camara, Baizhi Chen, An Qi Xu, Dinis Pedro Calado

**Affiliations:** 1 Immunity and Cancer Laboratory, The Francis Crick Institute, London, UK; 2 West African Centre for Cell Biology of Infectious Pathogens, University of Ghana, Accra, Ghana; 3 Immunity and Cancer Laboratory, The Babraham Institute, Babraham Research Campus, Cambridge, UK; 4 Immunology Programme, The Babraham Institute, Babraham Research Campus, Cambridge, UK

**Keywords:** Cell Biology, Immunology, Microscopy

## Abstract

Bone marrow plasma cells maintain durable antibody responses, but their tissue localization and morphology are difficult to quantify *in situ*. Here, we present a protocol for isotype-resolved 3D imaging and quantification of bone marrow plasma cells in murine femurs. We describe steps for fixation, optimal cutting temperature (OCT) coumpound embedding, longitudinal opening of mouse femurs, whole-mount immunostaining, optical clearing, and confocal 2D/3D imaging. We detail an Imaris-based workflow for reproducible single-cell segmentation, isotype-resolved counting, and the extraction of morphological features from defined regions of interest.

## Highlights

• Fix, cryoprotect, and OCT-embed mouse femurs for cryostat trimming
• Open femurs longitudinally for whole-mount immunostaining of marrow
• Clear and mount samples for confocal 2D tile scans and 3D z-stacks
• Segment isotype-resolved plasma cells and extract morphology in Imaris

## Abstract (editor highlights)

Publisher’s note: Undertaking any experimental protocol requires adherence to local institutional guidelines for laboratory safety and ethics.

## Graphical abstract

**Figure:**
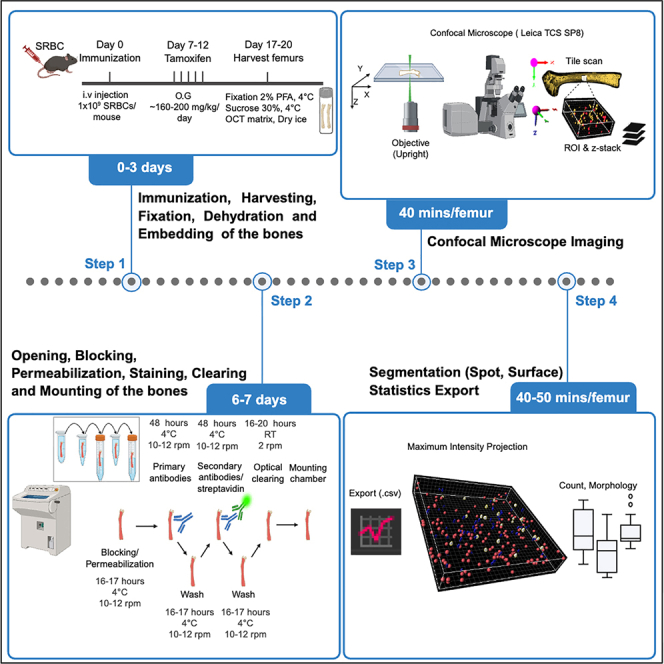

## Teaser

Bone marrow plasma cells maintain durable antibody responses, but their tissue localization and morphology are difficult to quantify *in situ*. Here, we present a protocol for isotype-resolved 3D imaging and quantification of bone marrow plasma cells in murine femurs. We describe steps for fixation, OCT embedding, longitudinal opening of mouse femurs, whole-mount immunostaining, optical clearing, and confocal 2D/3D imaging. We detail an Imaris-based workflow for reproducible single-cell segmentation, isotype-resolved counting, and the extraction of morphological features from defined regions of interest.

## Before you begin

Bone marrow (BM) plasma cells (PCs) support long-lived antibody titers by residing in specialized survival niches within the marrow microenvironment.^1^^,^^2^^,^^3^^,^^4^^,^^5^ BM PCs exhibit functional heterogeneity, including differential expression of immunoglobulin isotypes such as IgM, IgG, and IgA, each associated with distinct roles in immune responses. IgM^+^ PCs are linked to early or innate-like immune responses, IgG^+^ PCs to systemic, high-affinity responses, and IgA^+^ PCs to mucosal immunity. Current approaches to studying their biology rely on tissue dissociation and do not preserve spatial information, limiting our understanding of their biology within the BM microenvironment.

Direct imaging of PCs in intact bone is challenging because the mineralized matrix scatters light and many whole-bone clearing approaches can compromise antigenicity or impose substantial computational burden for segmentation.^6^^,^^7^^,^^8^ This protocol uses cryostat trimming to longitudinally open the femur and expose marrow on both faces, enabling whole-mount immunostaining, gentle optical clearing, confocal imaging, and reproducible Imaris-based analysis of isotype-defined PC subsets and morphology in situ.

Flow cytometry enables high-throughput characterization of PCs but requires tissue dissociation, resulting in loss of information about the native BM architecture. In contrast, the protocol described here preserves tissue integrity, enabling determination of isotype frequencies and relative numbers, as well as spatially resolved analysis of their localization and organization within the BM microenvironment.

The mouse model used in this protocol requires tamoxifen treatment to label PCs specifically via red fluorescent protein (RFP) expression driven by the *Jchain* promoter, which is selectively active in PCs and has been described.^9^ Thus, RFP expression marks PCs that have undergone Cre-mediated recombination at the time of tamoxifen induction.

### Innovation

Longitudinal opening of long bones has enabled whole-mount imaging of the marrow in prior works.^10^^,^^11^ This protocol provides a fully specified, end-to-end workflow optimized for plasma cell biology, combining isotype-resolved whole-mount immunostaining, confocal 2D/3D acquisition, and an Imaris analysis strategy that mirrors flow-cytometry-style gating to yield reproducible single-cell counts and morphology in intact bone marrow. The analysis is designed to be accessible to non-computational users while retaining transparent filtering criteria that can be tuned for dataset-specific signal-to-noise.

### Institutional permissions

All animal procedures outlined in this protocol were carried out at The Francis Crick Institute, where mice were housed and maintained under specific pathogen-free conditions. Experimental protocols involving animals received prior authorization from the institute’s Biological Resources Facility strategic oversight committee, including the Animal Welfare and Ethical Review Body, and were licensed by the UK Home Office in accordance with national regulations. Researchers intending to replicate this work must secure approval from their local institutional animal ethics committee and comply with all relevant institutional and governmental regulations. Proper safety protocols should be followed for the handling and disposal of hazardous materials, and a risk assessment must be conducted when required by local policy.

### Preparation of sheep red blood cells

**Timing: ∼ 50 min**

1. In a 50 mL Falcon tube:
  a. Add 5 mL of SRBC
  b. Add 45 mL of 1X PBS and mix
2. Centrifuge at 1800×*g* for 5 min at 20-25°C
3. Carefully aspirate the supernatant without disturbing the pellet
4. Resuspend the pellet in 50 mL of fresh:
  a. Repeat centrifugation at 1800×*g* for 5 min
  b. Ensure the supernatant is clear at this step

***Note:*** The supernatant should be clear at this step; if not, wash again and proceed with preparing a second tube.

5. Resuspend the washed SRBC pellet in approximately 3 mL of 1X PBS
6. Prepare serial dilutions for counting as follows:
  a. 10 μL of resuspended SRBCs
  b. Add 990 μL of 1X PBS and mix to prepare a 1:100 dilution
  c. Take 10 μL of 1:100 dilution
  d. Add 990 μL of 1X PBS and mix to achieve a final 1:10,000 dilution
7. Load the final 1:10,000 dilution into a clean hemocytometer chamber
8. Count the cell number according to the local facility

**CRITICAL:** Prepared SRBCs should not be refrigerated; administer to mice within 30-60 min post-preparation to preserve cell integrity and immunogenicity.

### Preparation of tamoxifen (40 mg/mL)

**Timing: ∼ 80 min**

9. Weigh 2 g of tamoxifen (Sigma-Aldrich)
  a. Transfer it to 50 mL Falcon tube
  b. Add 1 mL of 100% ethanol (2% of final volume)
  c. Mix gently at 20-25°C until fully dissolved
  d. Add 49 mL of sterile sunflower oil immediately to reach a final volume of 50 mL
  e. Wrap with foil
  f. Incubate at 37 °C for 1 h on a roller mixer or orbital shaker
10. Aliquot 2 mL into light-protected 2 mL Eppendorf tubes

***Note:*** The aliquots should be stored at - 20 °C for up to one year. Avoid repeated freeze-thaw cycles.

## Key resources table

**Table:** 

REAGENT or RESOURCE	SOURCE	IDENTIFIER
**Antibodies**		

Rat anti-mouse endomucin (Clone V.7C7), unconjugated (dilution: 1:200)	Santa Cruz Biotechnology, Inc.	Cat#sc-65495; RRID:AB_2100037
Goat anti-rat Alexa Fluor 488 (dilution: 1:200)	Thermo Fisher Scientific	Cat#A11006; RRID:AB_2534074
Rabbit anti-RFP Antibody Pre-adsorbed (dilution: 1:200)	Rockland Immuno chemicals Inc	Cat#600-401-379; RRID:AB_2209751
Donkey anti-rabbit IgG (H+L) Highly Cross-Adsorbed (dilution: 1:200)	Fisher Scientific Ltd	Cat#10749004
Goat anti-mouse IgM μ chain Alexa Fluor 405 (dilution: 1:100)	Abcam	Cat#ab175662; RRID:AB_2920723
Goat anti-mouse IgA alpha chain Biotin (dilution: 1:100)	Abcam	Cat#ab97233; RRID:AB_10681185
PE anti-mouse CD138 (Syndecan-1) Antibody (dilution: 1:50)	Biolegend	Cat#142504; RRID:AB_10916119
Goat anti-Mouse IgG, IgM, IgA (H+L) Cross-Adsorbed Secondary Antibody, Biotin (dilution: 1:500)	Thermo Fisher Scientific	Cat#A-10676; RRID:AB_2534059
Goat anti-mouse IgM μ chain Alexa Fluor 488 (dilution: 1:100)	Abcam	Cat#ab150121; RRID:AB_2801490

**Biological samples**		

Sheep Blood Defibrinated −100ml	TCS Biosciences	Cat#SB054

**Chemicals, peptides, and recombinant proteins**		

Tamoxifen	Sigma-Aldrich	Cat#T5648
Sunflower seed oil	Sigma-Aldrich	Cat# S5007-250ml
16% formaldehyde (w/v), Methanol-free (dilution: 2% in 1X PBS)	Thermo Fisher Scientific	Cat#28908
Sucrose (dissolved at 30% in 1X PBS)	Merck Life Science UK Limited	Cat#S9378-500G
OCT embedding matrix - 125ml	CellPath	Cat#KMA-0100-00 A
Gibco Phosphate-Buffered Saline (1X PBS), pH 7.4	Thermo Fisher Scientific	Cat#10010023
Triton X-100 (dilution: 0.25% in 1X PBS)	Sigma-Aldrich	Cat# X100-500ML
Bovine Serum Albumin (dissolved at 1% in 1X PBS)	Sigma-Aldrich	Cat# A2153-100G
Donkey serum (dilution: 10% in 1X PBS)	Sigma-Aldrich	Cat#D9663-10ML
Streptavidin, Alexa Fluor 647 Conjugate (dilution: 1:500)	Thermo Fisher Scientific	Cat#S21374
DAPI (1:1000 in 1X PBS)	Sigma-Aldrich	Cat#MBD0015-5ML
RapiClear® 1.52	SunJin Lab	Cat#RC152001

**Experimental models: Organisms/strains**		

Mouse: Jchain-CreERT2; Rosa26 reporter mice: Jchaintm1(EGFP/cre/ERT2)Wtsi); Gt(ROSA)26Sortm1Hjf (C57BL/6 J background) Male and female (8-12 weeks)	Xu et al.^9^	MGI:5633773 MGI:3696099

**Software and algorithms**		

Leica Application Suite X (LAS X)	Leica Microsystems	https://www.leicamicrosystems.com
Imaris 10.2.0 or higher	Oxford Instruments	https://imaris.oxinst.com
Imaris File Converter 10.2.0	Oxford Instruments	https://imaris.oxinst.com
GraphPad Prism version 10.6.1 (799)	GraphPad Software	https://www.graphpad.com/company

**Other**		

15 mL and 50 mL Falcon tubes	Thermo Fisher Scientific	Cat#339651 Cat#339653
2 mL Eppendorf Safe-Lock tubes	Eppendorf	Cat#0030120094
1 mL Eppendorf Safe-Lock tubes	Eppendorf	Cat#0030120086
5 mL disposable bijou container	SciQuip Ltd	Cat#500-1000-417
Petri dish (100 -150 mm diameter)	VWR International	Cat#391-0850
0.22 μm syringe filters (33 mm)	Merck	Cat#SLGPM33RS
0.22 μm syringe filters (13 mm)	Merck	Cat#WHA10462945
1 mL, 10 mL, 20 mL syringes	BD PlastiPak™	Cat#15489199 Cat#15544835 Cat#10569215
1 mL Insulin Syringe, 26G needle	Fisher Scientific Ltd	Cat#17132714
Disposable scalpels	Swann-Morton	Cat#0501
MX35 Ultra Microtome Blade	Thermo Fisher Scientific	Cat#3053835
Single-edge razor blade	SciQuip Ltd	Cat#AGT586
Sharp bent round forceps	Fisher Scientific Ltd	Cat#10654101
Scissors SS pointed 160 mm long	VWR International	Cat#233-1212
Disposable Base Mold, 24 x 24 x 5 mm (Cyromold)	Clinisciences Limited Or Scientific Laboratory Supplies	Cat#25376-500 Cat#HIS0222
iSpacer 3mm (Rectangular well)	SunJin Lab	Cat#IS013
Cover Glass 22x32 mm Thickness No.1.5	VWR International	Cat# 631-0134
Roller mixer (12 rpm)	Wolf Laboratories Limited	Cat#MXS-10
Rotating Mixer (2 rpm)	Stuart	Cat#SB3
Leica CM3050 S Cryostat	Leica Biosystems	Cat#CM3050 S
Leica TCS SP8 Confocal Microscope, Microscope body (Leica DMi8), Objective 20× HC PL APO CS2 dry, Laser lines (405 nm, 488 nm, 561 nm, 633 nm), Software module (LAS X Spiral Tile Scan), z-stack imaging (z-depth: ∼ 60 μm, Resolution: 512 × 512), Image stitching (Merge function of LAS X software).	Leica Microsystems	TCS SP8

## Materials and equipment

### Microscope and software

• Confirm that your confocal microscope can acquire stitched 2D tile scans and 3D z-stacks (e.g., LAS X Navigator/Spiral or equivalent).
• Install Imaris (v10.2.0 or later) and Imaris File Converter; confirm that statistics can be exported to.csv and that downstream analysis can be performed in GraphPad Prism.
• **Phosphate buffered saline (1X PBS)**

We used commercially available 1X PBS (pH 7.4; Gibco, Cat#10010023) throughout the protocol to ensure reproducibility and cleanliness.

• **Fixation buffer (2%)**

Dilute 16% methanol-free formaldehyde stock 1:8 in sterile 1X PBS. Prepare fresh on the day of use.

**CRITICAL:** Formaldehyde is a dangerous chemical. Handle and discard with caution as outlined by the product safety data sheet and institutional guidance.

• **Sucrose (30%)**.

Dissolve 30 g of sucrose in 100 mL of 1X PBS. Prepare fresh on the day of use.

• **Blocking/Permeabilization Buffer**.

The final volume below is enough for up to 5 femurs (2 mL of buffer per femur).

**Table:** 

Reagent	Final concentration	Amount
Donkey serum	10% (v/v)	1 mL
BSA	1% (w/v)	100 mg
TritonX100	0.25% (v/v)	25 μL
1X PBS	N/A	to 10 mL
**Total**	**N/A**	**10 mL**

***Note:*** The Blocking/Permeabilization Buffer must be filtered through a 0.22 μm filter, stored at 4 °C, and used within 20-30 h of preparation.

**CRITICAL:** Triton X-100 is highly viscous, making it difficult to pipette small volumes accurately. Cut the pipette tip before dispensing, and prepare ≥10 mL of total buffer to ensure the Triton X-100 volume exceeds 20 μL for improved accuracy.

• **Primary Antibody Cocktail**

The final volume (1 mL) presented here is informative for 1 femur.

**Table:** 

Reagent	Final concentration	Amount
Rabbit anti-RFP Antibody Pre-adsorbed (600-401-379)	1:200	5 μL
Rat anti-mouse endomucin (V.7C7): sc-65495	1:200	5 μL
Goat anti-mouse IgM μ chain (Alexa Fluor 405 (ab175662)	1:100	10 μL
Goat Anti-Mouse IgA alpha chain Biotin, (ab97233)	1:100	10 μL
Blocking/Permeabilization buffer	N/A	970 μL
**Total**	**N/A**	**1000 μL**

***Note:*** The Primary Antibody Cocktail must be prepared fresh and used immediately.

• **Secondary Antibody Buffer**

The final volume is indicative and sufficient for 10 femurs (1 mL per femur).

**Table:** 

Reagent	Final concentration	Amount
Donkey serum	10% (v/v)	1 mL
TritonX100	0.25% (v/v)	25 μL
1X PBS	N/A	to 10 mL
**Total**	**N/A**	**10 mL**

***Note:*** This buffer is prepared to make the Secondary Antibody Cocktail and should not contain BSA.

• **Secondary Antibody Cocktail**

**Table:** 

Reagent	Final concentration	Amount
Donkey anti-rabbit IgG (H+L) Highly Cross-Adsorbed Alexa Fluor 555 (10749004)	1:200	5 μL
Goat anti-rat Alexa Fluor 488 (A11006)	1:200	5 μL
Streptavidin, Alexa Fluor™ 647 Conjugate (S21374)	1:500	2 μL
Secondary Antibody buffer	N/A	988 μL
**Total**	**N/A**	**1000 μL**

***Note:*** The Secondary Antibody Cocktail must be prepared fresh and used immediately.

• **Clearing solution**

RapiClear 1.52 (Cat# RC152001, SunJin Lab) is used for optical clearing and refractive index matching of the femur with the glass (Cat# 631-0134). It is a ready-to-use, glycerol-based proprietary clearing reagent.

***Note:*** RapiClear is considered a low-hazard reagent compared to solvent-based clearing agents. Handle with appropriate PPE (lab coat, gloves) and dispose of waste according to institutional guidelines.

• **Microscope detection windows**

**Table:** 

Fluorophore	Excitation laser (nm)	Emission detection window (nm)
Alexa Fluor 405	405	412–464
Alexa Fluor 488	488	504–548
Alexa Fluor 555	553	558–614
Alexa Fluor 647	653	658–775

## Step-by-step method details

### Mouse immunization, treatment, and femur fixation

**Timing: 0–3 days after harvesting femurs**

This section describes immunization, tamoxifen-induced plasma cell labelling, femur harvesting, and fixation prior to downstream processing.

1. Immunize mice with SRBCs
  a. Prepare SRBCs at 5 × 10^9^ cells/mL in sterile 1X PBS
  b. Inject 200 μL of the SRBC intravenously via the tail vein of adult mice

***Note:*** This corresponds to 1x10^9^ SRBCs injected per mouse. This regimen is widely used as a gold standard for eliciting robust, T-dependent B cell responses, including germinal center formation, isotype switching, and plasma cell differentiation.^9^

2. Administer tamoxifen to induce plasma cell labelling
  a. Remove tamoxifen from −20 °C storage and thaw by gently warming the tube in your hand until the solution is fully liquefied.
  b. Administer 100 μL per mouse per day by oral gavage for five consecutive days
  c. Start treatment on day 7 post-immunization

***Note:*** This corresponds to 160-200 mg/kg/day of tamoxifen in 8-12-weeks-old mice. For older (heavier) mice, adjust the volume proportionally to maintain the same dose per kilogram of body weight.

3. Cull the mice 5 days post-tamoxifen.
  a. Place the mouse in a supine position on a dissection board.
  b. Sterilize the skin with 70% ethanol.
  c. Use surgical scissors to make an incision starting at the knee joint up toward the lower abdomen.
  d. Cut around the thigh to fully expose the hindlimb and.
  e. Peel the skin away to reveal the underlying musculature.
  f. Remove as much muscle tissue as possible using scissors.
  g. Dislocate the hip joint to expose the full length of the femur.
  h. Cut above the hip joint and below the knee joint to release the femur.
4. Fix the harvested femurs.
  a. Immediately transfer the femurs into 5 mL ice-cold 2% formaldehyde in 1X PBS
  b. Place two femurs per tube

Incubate at 4°C for 16-17 h to ensure adequate penetration into the dense bone marrow compartments.

***Note:*** Ensure bones are thoroughly cleaned of muscle tissue for adequate fixation. Place in ice-cold 1X PBS, then use forceps and a scalpel to carefully remove any remaining tissue before transferring to 2% formaldehyde. Shaking during fixation is not required.

**CRITICAL:** Higher formaldehyde concentrations (4%) may increase background artefacts and reduce fluorescence. Notably, the RFP signal was weak at 4% formaldehyde. We found that 2% formaldehyde provides an optimal balance between antigen preservation and image quality.

5. Wash the fixed femurs.
  a. Transfer femurs to a fresh tube containing 5 mL of 1X PBS.
  b. Incubate at 20–25°C for 10 min on a roller mixer.
  c. Discard the PBS and repeat the wash with 5 mL of 1X PBS.
6. Dehydrate the femurs for cryoprotection
  a. Transfer the femurs to a new tube containing 5 mL of 30% sucrose in 1X PBS
  b. Incubate at 4°C for 2 days.

***Note:*** This protocol does not require bone decalcification. 30% sucrose was used to dehydrate the tissue after fixation, before freezing. This step prevents ice crystal formation and preserves bone marrow architecture.

### Bone embedding

**Timing: 5–10 min per femur**

This section describes embedding fixed femurs in OCT compound to enable cryostat sectioning and expose bone marrow.

**Figure 1:**
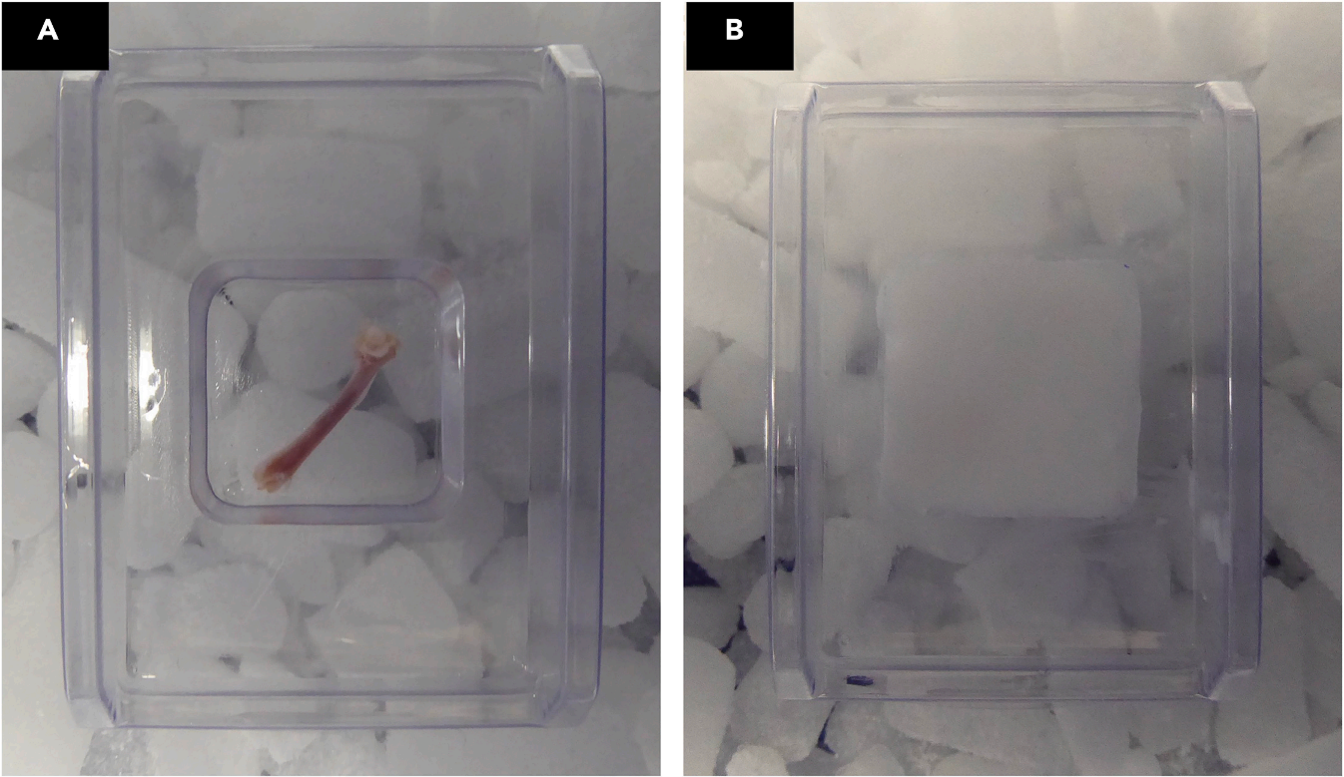
Embedding the femur into the OCT for freezing (A) The image of the diagonal positioning of the femur in OCT before freezing on dry ice. (B) The image of the frozen OCT block containing the femur. Images were taken with a Panasonic DMC-TZ70 camera.
  e. Add additional OCT compound to fully cover the femur

***Note:*** Avoid creating bubbles while pouring the OCT matrix. If bubbles form, gently remove them using a pipette tip.

7. Prepare a 24 × 24 × 5 mm-sized cryomold on a flat surface
  a. Fill the cryomold with OCT embedding matrix
  b. Place each femur into the OCT-filled mold
  c. Position the femur diagonally along the bottom of the mold
  d. Ensure that both ends of the femur are aligned horizontally (Figure 1A).

8. Freeze the embedded samples
  a. Place the mold on dry ice until the OCT is fully solidified (Figure 1B)
  b. Remove the mold from the dry ice
  c. Gently rub the bottom with your finger for 10–15 seconds to loosen the block slightly
  d. Carefully push the block out with your thumb to release it
  e. Immediately wrap it in aluminium foil, then place it back on dry ice

***Note:*** The frozen OCT block can be stored at −80°C for up to a year before processing for imaging.

### Longitudinal bone opening

**Timing: 10–20 min per femur**

This section describes cryostat trimming of OCT-embedded femurs to expose the bone marrow cavity in a longitudinal orientation for whole-mount staining.

9. Set cryostat temperatures
  a. Set the cryostat chamber to −20°C and the sample holder to −17°C
  b. Allow the cryostat to reach those temperatures before introducing the samples
10. Mount the OCT block.
  a. Place the block onto the cryostat specimen holder using a small amount of OCT as adhesive
  b. Ensure the longitudinal axis of the femur is aligned parallel to the cutting direction (Figure 2A)

**Figure 2:**
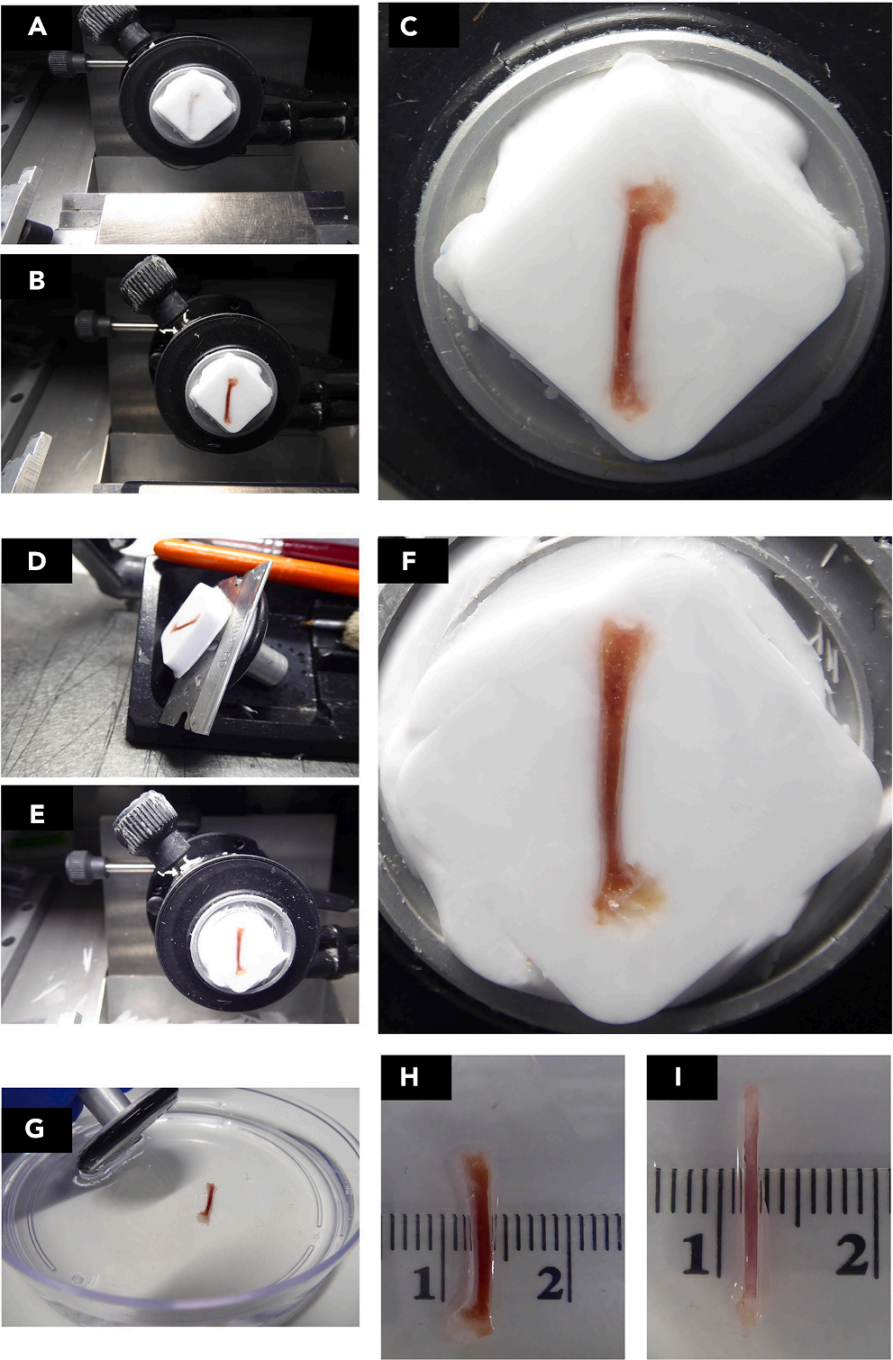
Step-by-step cryostat trimming and OCT removal to open the femurs and expose the bone marrow (A) The OCT-embedded femur is mounted vertically onto the cryostat chuck for sectioning. (B and C) The bone is trimmed until the BM cavity is visible (appearing red). (D) The block is removed, flipped, and remounted with the previously exposed side affixed to the chuck to allow trimming from the opposite end. (E and F) The second trimming exposes the BM cavity on both ends, resulting in an open, longitudinal bone slice. (G) The chuck with the attached block is placed gently onto a Petri dish containing 1X PBS. The OCT embedding medium dissolves in 1X PBS, leading to spontaneous detachment of the femur slice. (H) Medial view showing the exposed marrow of the opened femur. (I) Lateral view showing bone matrix surrounding the exposed marrow. Images were taken with a Panasonic DMC-TZ70 camera.
11. Expose the bone marrow cavity.
  **CRITICAL:** The femur must be oriented vertically during sectioning. Trimming in a horizontal orientation can compress the bone marrow and distort the structure.
  a. Trim the block to a thickness of 20–30 μm to remove excess OCT
  b. Reduce trimming thickness to 8–10 μm as the blade reaches the cortical bone.
  c. Continue until the entire bone marrow cavity is exposed (Figures 2B and 2C)
  d. Remove the specimen holder with the block still attached, carefully
  e. Detach the block with a single-edge razor blade (Figure 2D)
  f. Flip the block and remount it so the opposite side can be sectioned
  g. Trim again until the bone marrow cavity is exposed on the opposite face (Figures 2E and 2F)
    ***Note:*** Because the bone is embedded in OCT, which provides mechanical support, a cortical bone fracture is unlikely. However, once the BM is exposed, trim gently at a lower thickness (8–10 μm) to ensure smooth contact with the BM.
    **CRITICAL:** Ensure the cryostat blade (MX35 Ultra Microtome Blade) is not dented, as this may damage the structure of the exposed BM.
12. Transfer the opened femur slices
  a. Remove the specimen holder with the block
  b. Gently submerge the block in 1X PBS in a Petri dish
  c. Gently shake to dissolve the OCT compound and release the bone (Figure 2G)
    ***Note:*** While shaking, avoid touching the bottom of the Petri dish to preserve the integrity of the exposed bone marrow.
  d. Using forceps, gently grasp one of the femoral heads (epiphysis)
  e. Avoid holding the shaft (diaphysis) to prevent damage to the exposed marrow
  f. Transfer the sample to 5 mL of 1X PBS in a bijou container
  g. Place the tube on a roller mixer set to low speed (10–12 rpm)
  h. Incubate at 20–25°C for 5 min
  i. Discard the PBS and repeat steps (f-h) to remove any residual OCT
    **CRITICAL:** For the bone imaged in this protocol, the thickness was ∼1 mm (Figure 2I). The more you trim, the thinner the resulting bone slice will be. Adjust the trimming depth to suit your experimental needs.

### Immunostaining for isotype labeling of plasma cells

**Timing: 8–10 days**

This section describes whole-mount immunostaining of longitudinally opened femurs to detect isotype-defined plasma cells and vascular structures.

**Figure 3:**
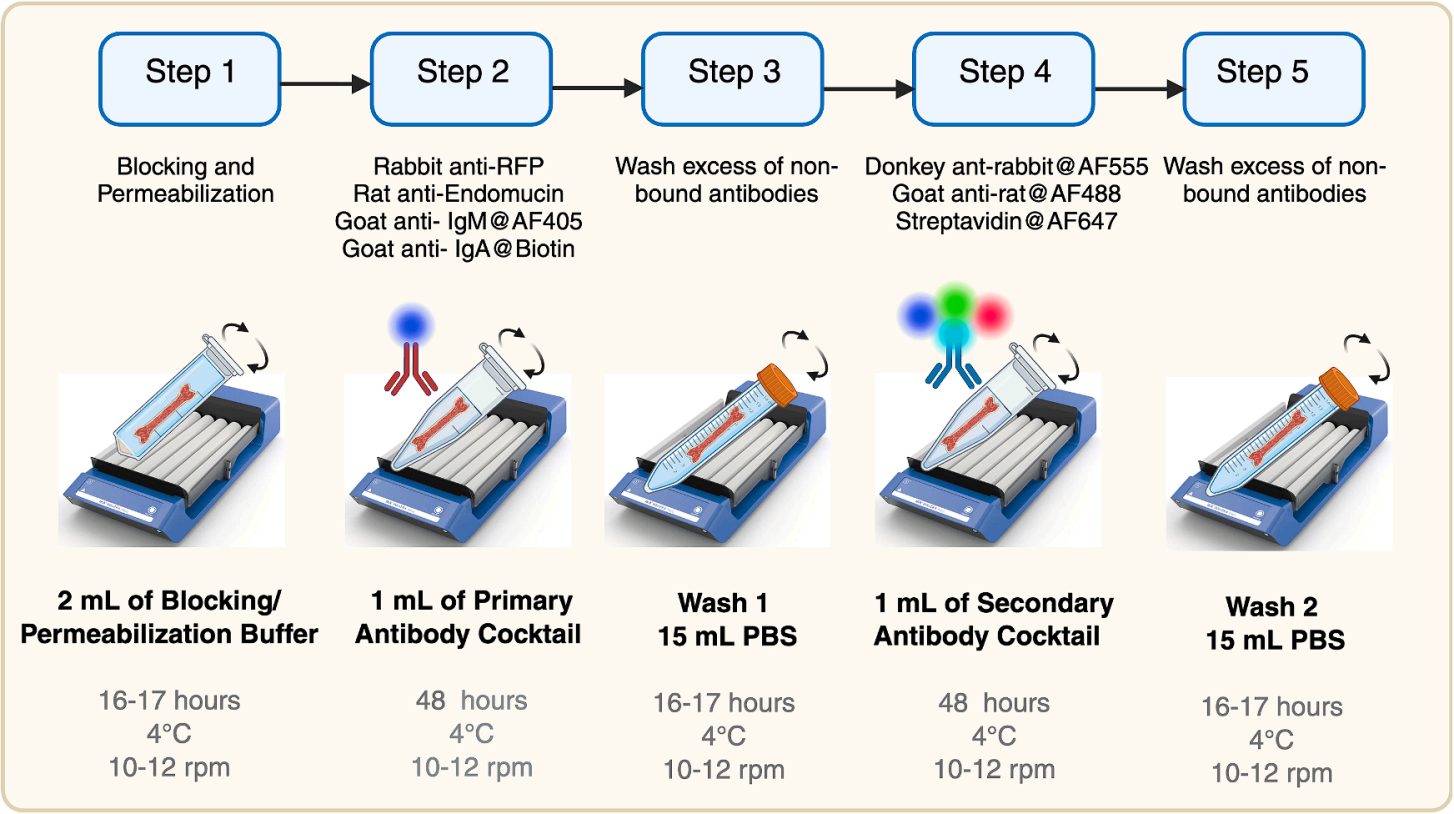
Workflow of Immunostaining for isotype-specific plasma cells Step 1: Blocking and permeabilization. Femur is incubated in 2 mL of Blocking/Permeabilization buffer for 16 -17 h at 4°C under rotation at 10-12 rpm. Step 2: Primary antibody incubation. Femur is incubated for 48 h at 4°C, under rotation at 10-12 rpm in 1 mL of freshly prepared Primary Antibody Cocktail containing rabbit anti-RFP, rat anti-Endomucin, goat anti-IgM Alexa Fluor 405, and goat anti-IgA biotin. Step 3: First wash. Excess primary antibodies are removed by washing in 15 mL 1X PBS for 16 - 17 h at 4°C, under rotation at 10-12 rpm. Step 4: Secondary antibody incubation. Femur is incubated for 48 h at 4°C, under rotation at 10-12 rpm in 1 mL of Secondary Antibody Cocktail containing anti-rabbit Alexa Fluor 555, anti-rat Alexa Fluor 488, and streptavidin Alexa Fluor 647. Step 5: Second wash. Excess secondary antibodies are removed by washing in 15 mL 1X PBS for 16 - 17 h at 4°C, under rotation at 10-12 rpm. RFP^+^ cell is visualized in red (AF555), vasculature in green (AF488), IgM^+^ plasma cells in blue (AF405), and IgA^+^plasma cells in far-red (AF647).
  b. Incubate at 4°C for 16–17 h on a roller mixer set to 10–12 rpm

***Note:*** Ensure the sample is fully submerged in buffer to allow uniform reagent penetration.

13. Block and permeabilize the sample
  a. Transfer each opened femur to a 2 mL Eppendorf Safe-Lock tube containing 2 mL of Blocking/Permeabilization buffer (Figure 3, Step 1)

14. Incubate with primary antibodies
  a. Transfer the femur to a new 1.5 mL Eppendorf Safe-Lock tube containing 1 mL of Primary Antibody Cocktail (see Figure 3, Step 2)
  b. Incubate at 4°C for 48 h on a roller mixer at 10–12 rpm

**CRITICAL:** Ensure gentle mixing throughout incubation to achieve uniform antibody distribution within the tissue

15. Wash after primary antibody incubation
  a. Transfer the femur to a 15 mL tube containing 15 mL of 1X PBS (Figure 3, Step 3).
  b. Incubate at 4°C for 16–17 h on a roller mixer
16. Incubate with secondary antibodies.
  a. Transfer the femur to a new 1.5 mL tube containing 1 mL of the Secondary Antibody Cocktail (Figure 3, Step 4)
  b. Incubate at 4°C for 48 h on a roller mixer at 10–12 rpm
17. Perform final wash.
  a. Transfer the femur to a 15 mL tube containing 15 mL of 1X PBS (Figure 3, Step 5)
  b. Incubate at 4°C for 16–17 h on a roller mixer.

### Optical clearing

**Timing: 16–20 h**

This section describes the optical clearing of immunostained femurs using RapiClear to enhance light penetration for high-resolution confocal imaging.

18. Prepare the clearing reagent
  a. Remove RapiClear from −20°C storage before use
  b. Warm it to 37°C using a water bath or an incubator

***Note:*** Warming the RapiClear solution reduces its viscosity and improves tissue penetration

**Figure 4:**
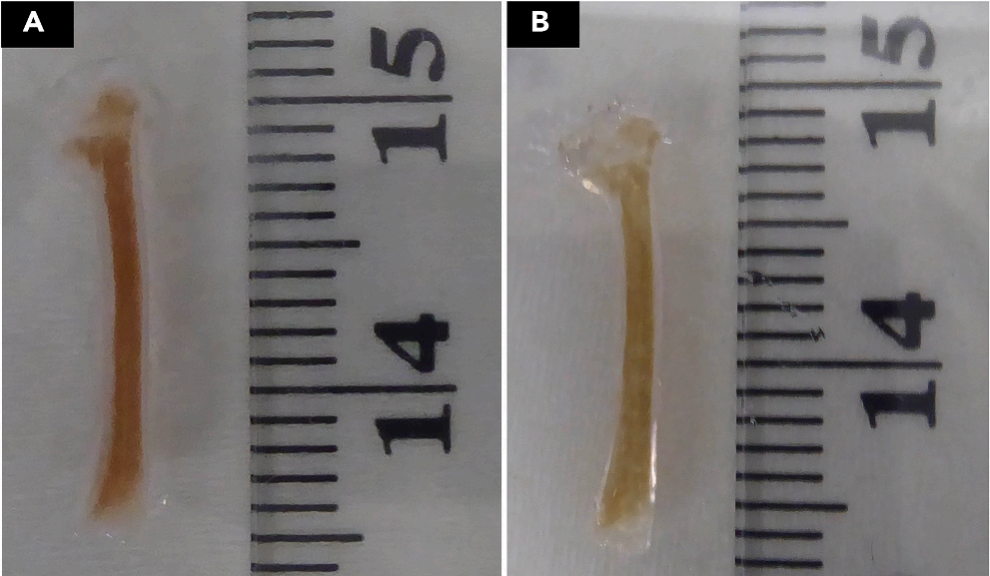
Image of an open femur Before (Left, A) and after (Right, B) clearing.

19. Transfer the femur to clearing solution.
  a. Place each stained femur in a 1.5 mL Eppendorf Safe-Lock tube
  b. Add 1 mL of RapiClear to fully immerse the sample
    ***Note:*** Protect from light with foil and ensure the femur is fully submerged to achieve uniform refractive index matching.
  c. Place the tube on a rotating mixer set to 2 rpm
    **CRITICAL:** Adjust the rotating mixer to 2 rpm/min to ensure the femur moves up and down through the RapiClear solution with each 180° rotation. This motion enables efficient and uniform penetration of the clearing agent into the tissue.
  d. Incubate at 20–25°C for 16–20 h
20. Confirm the clearing efficiency
  a. Inspect the femur visually for increased transparency
  b. Compare appearance before and after clearing if necessary (see Figure 4)

### Mounting the cleared bone for imaging

**Timing: 5–10 min per femur**

This section describes the preparation of an imaging chamber using an iSpacer and the mounting of the cleared femur for stable confocal acquisition.

**Figure 5:**
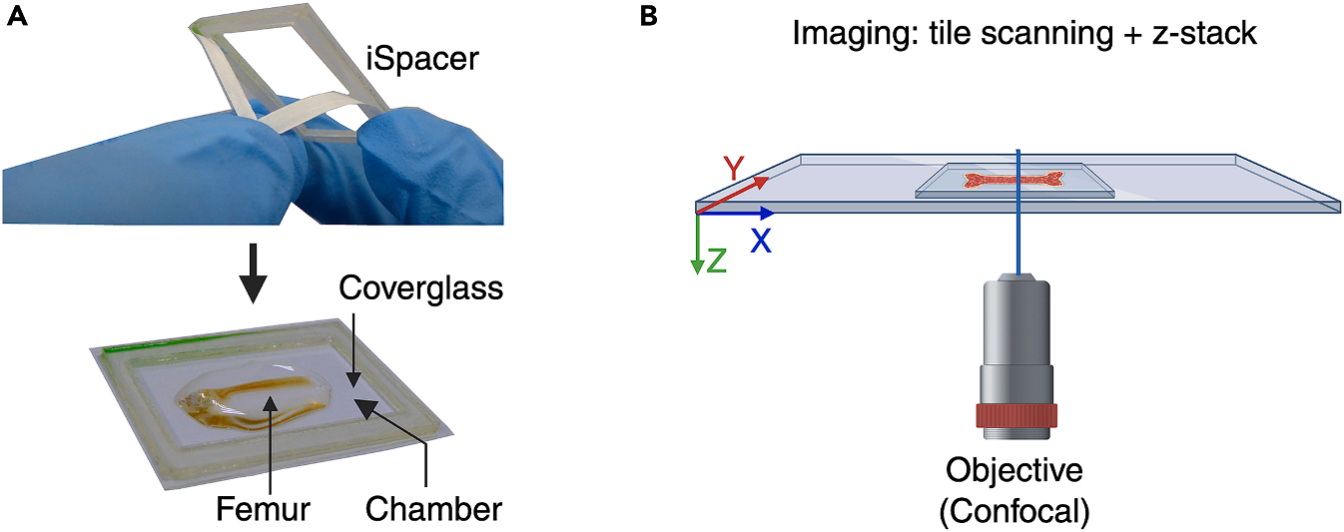
Mounting the clear femur and positioning it to the microscope objective for imaging (A) Assembly of the imaging chamber using a 3 mm iSpacer adhered to a coverglass (22 × 32 mm, No. 1.5). The femur is placed centrally within the chamber and immersed in RapiClear. (B) Confocal imaging setup showing the correct bone orientation. The femur is aligned parallel to the X-axis of the microscope stage to enable tile scanning in the XY plane and z-stack acquisition up and down along the z-axis.
  b. Align the adhesive side of the iSpacer with a clean coverslip (22 × 32 mm, No. 1.5 thickness)

***Note:*** This coverslip serves as the base of the imaging chamber.

21. Prepare the imaging chamber using the iSpacer (IS013)
  a. Peel off the protective film from one side of the iSpacer to reveal the adhesive surface (Figure 5A, Top)

22. Position the cleared femur in the chamber
  a. Using forceps, gently transfer the cleared femur into the center of the chamber (Figure 5A, Bottom)
  b. Position it so that the diaphysis is parallel to the longer side of the iSpacer

**CRITICAL:** This orientation is essential for optimal imaging, as most confocal microscopes scan in the XY plane, as illustrated in Figure 5B. This alignment ensures that the entire length of the femur is captured within the imaging field before the z-stack acquisition of different regions of interest.

23. Add Rapiclear to the chamber.
  a. Add RapiClear dropwise to the chamber until the femur is fully immersed. (Figure 5A, Bottom)

**CRITICAL:** Avoid adding excessive RapiClear, as this may cause the femur slice to float. Movement during imaging can lead to misalignment, compromising image quality and downstream processing.

24. Mount the femur on the microscope stage
  a. Turn on the Leica TCS SP8 Confocal Microscope according to your local instructions
  b. Place the imaging chamber onto the microscope stage (see Figure 6)

**Figure 6:**
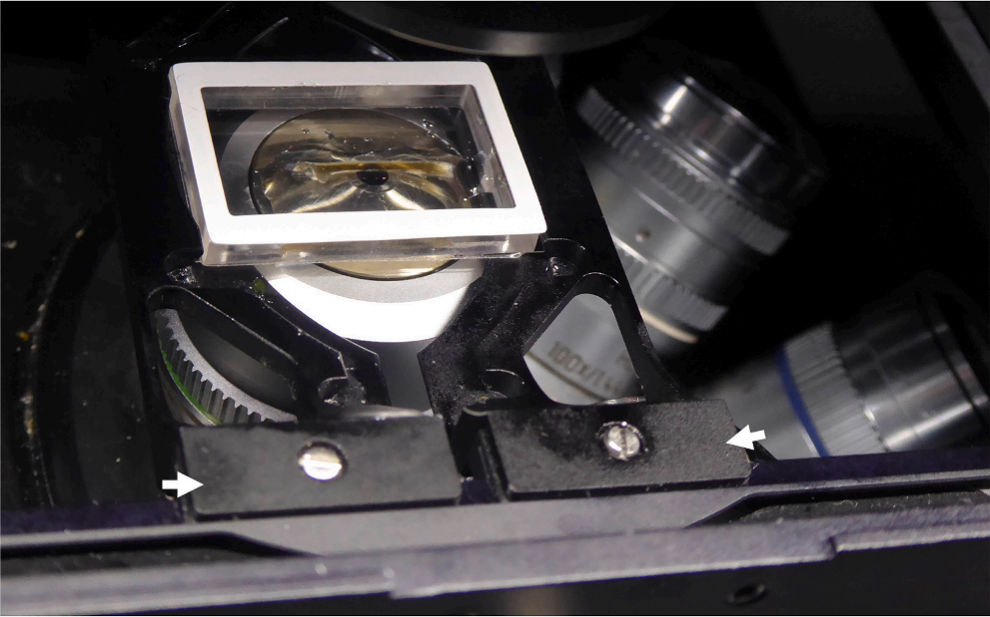
Positioning the imaging chamber with the femur at the center and embedded in RapiClear The arrows indicate clipping of the stage clips.
  c. Secure the chamber with stage clips to prevent movement (see Figure 6, arrows)
25. Position the femur.
  a. Use the stage controls or the joystick to center the femur under the objective
  b. Raise the objective to a working distance of approximately 2-3 mm

**CRITICAL:** Ensure the objective does not hit the coverslip brutally to avoid damage to both the sample and the microscope.

### 2D tile scan and 3D z-stack imaging of the bone

**Timing: 30–40 min per femur**

This section describes the configuration of the confocal microscope, acquisition of whole-bone 2D tile scans, and selection of regions of interest (ROIs) for 3D z-stack imaging.

26. Launch the imaging system
  a. Open the software LAS X by double-clicking on the icon
  b. Navigate to the Configuration tab (Figure 7 → 1)

**Figure 7:**
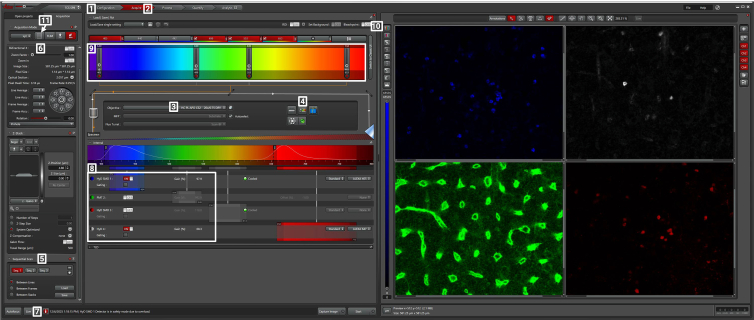
Leica Application Suite X (LAS X) interface and multichannel confocal imaging setup 1: Configuration tab, 2: Acquire tab, 3: Objective selection, 4: Fluorophore/beam path panel, 5: Sequential scan control panel, 6: Scan mode control panel, 7: Live preview, 8: Detector panel showing active HyD/PMT detectors and Gain (%) settings for each fluorophore, 9: Channel spectral layout and laser power settings for Alexa 405, Alexa 488, Alexa 555, and Alexa 647, 10: Range indicator for pixel saturation view.
  c. Set the acquisition bit depth to 16-bit
    **CRITICAL:** Always confirm and set the bit depth to 16-bit each time you open the software. Using a lower bit depth will reduce image quality.
  d. Go back to Acquire (Figure 7 → 2).
  e. Choose the 20×/0.75 DRY objective from the Touchscreen Control Panel of the microscope stand.
  f. Press FLUO and open the shutter.
  g. Use the endomucin (green) signal to bring the sample into focus.
  h. Close the shutter once the focus is set.
  i. Confirm that 20×/0.75 DRY objective is selected (Figure 7 → 3), from the software interface.
27. Configure the beam path using the Dye Assistant (Figure 7 → 4).
  a. Select Dyes: ALEXA 405, ALEXA 488, ALEXA 555 and ALEXA 647.
  b. Identify the dye combination with no crosstalk.
  c. Set the scan mode to Frame or Stack Sequential with 3 sequences.
  d. APPLY.

**CRITICAL:** This beam path configuration uses three sequential scans to eliminate spectral overlap. Sequence 1: Alexa 405 (blue) and Alexa 647 (far-red). Sequence 2: Alexa 488 (green). Sequence 3: Alexa 555 (yellow/orange). This arrangement eliminates crosstalk and ensures clean spectral separation across all fluorochromes.

**Figure 8:**
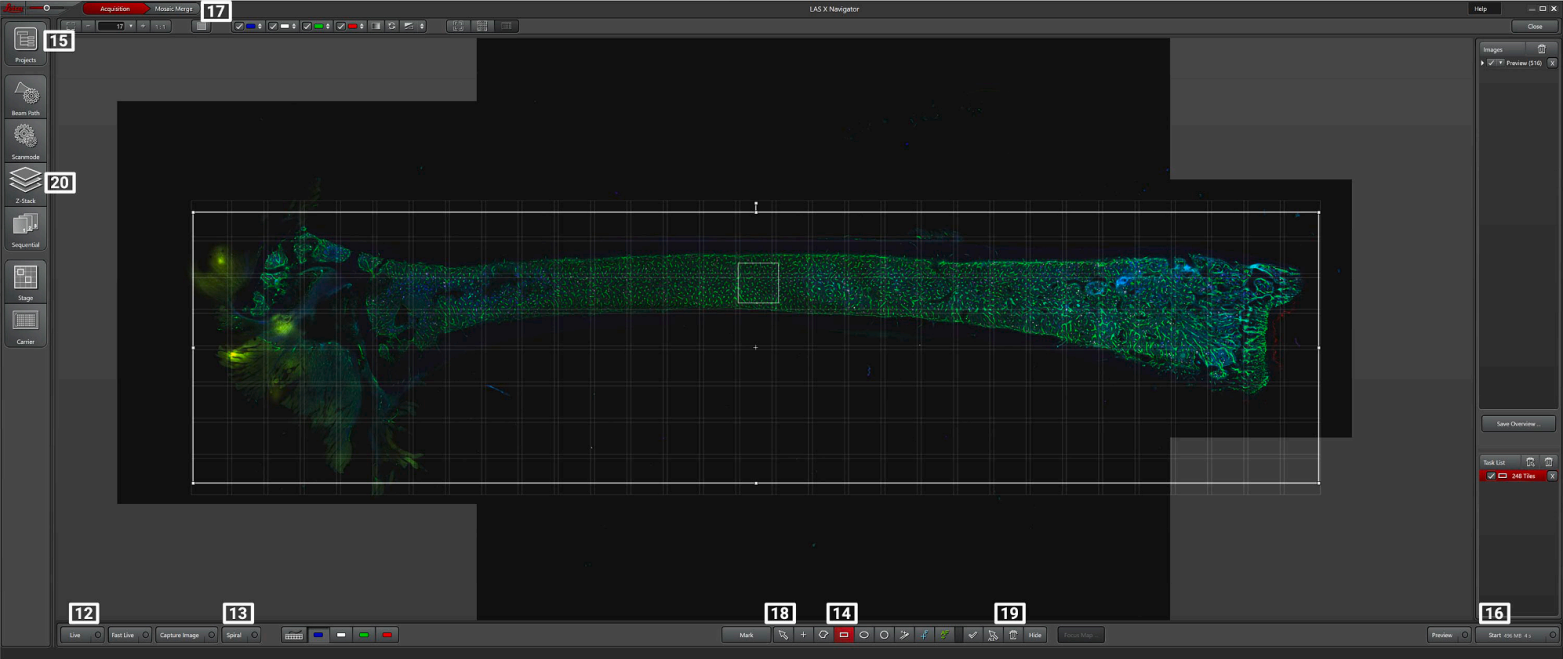
Navigator interface, Spiral autofocus, and Tile scan setup for entire femur slice Imaging 12: Live mode, 13: Spiral autofocus brings an entire slide view of the femur for region selection (Rectangle) for tile scanning, 14: Rectangle tool used to define the tile scan region, 15: Project creation panel,16: Start button used to begin the tile scan of the displayed and selected region, 17: Mosaic Merge interface for stitching the acquired tiles and selecting the tile-scanned image and leaving Blend as Smooth generates a 2D composite image of the entire femur, 18: Select the rectangle of interest, 19: delete the selected rectangle, 20: z-stack menu.

***Note:*** Spiral will automatically search for focus by moving in a spiral pattern to scan and display the entire femur. This may take about 10 min.

28. Set scan parameters
  a. Select between lines in Sequential Scan (Figure 7 → 5)
  b. Use the following settings for Scan mode (Figure 7 → 6)
    i. Format: 512 x 512
    ii. Scan speed: 400
    iii. Image size: 581.25 μm x 581.25 μm
    iv. Pixel size: 1.14 μm x 1.14 μm
    v. Optical section: 2.057 μm
29. Start live acquisition
  a. Click Live to start the live preview (Figure 7→ 7)
    ***Note:*** The four-channel preview is displays on the right.
  b. Open Sequence 1 and adjust the settings for Alexa 405 and Alexa 647 as below:
    i. Ensure that HyD SMD1 (Alexa 405) and HyD4 (Alexa 647) are switched ON (Figures 7→ 8)
    ii. Increase respective laser powers (Figures 7→ 9) slowly until labelled structures become visible
    iii. Click on range indicator view (Figures 7→ 10)
    iv. Adjust the Gain slider (Figures 7→ 8) until the signal is clear without saturating pixels
      ***Note:*** Avoid blue “overexposed” indicating pixel saturation.
  c. Switch to Sequence 2:
    i. Ensure PMT 2 is ON
    ii. Increase laser power and gain until vascular staining (green) appears without saturation
  d. Switch to Sequence 3:
    i. Ensure HyD SMD3 detector is ON
    ii. Increase laser power and gain until plasma cells (Red) appear without saturation
      ***Note:*** The display color of each fluorochrome in the live preview can be changed by clicking on its assigned color box (Figure 7→ 8).
      ***Note:*** A sequence of the step is
30. Acquire 2D tile scan of the entire femur
  a. Click the grid icon to launch the Navigator interface (Figure 7→ 11)
  b. Select Live mode (Figure 7→ 12)
  c. Activate Spiral search to automatically scan the sample (Figure 8 → 13)

**CRITICAL:** If the femoral heads do not appear as shown in Figure 8 at the end of the first Spiral, double-click near each head and re-enable Spiral.

31. Define the tile scan region
  a. Select the rectangle tool with a simple click (Figure 8 → 14)
  b. Draw a region covering the entire femur by left-clicking and holding while dragging
    ***Note:*** The rectangle can be resized by pulling its corner handles.
  c. Open Project (Figure 8 → 15)
  d. Name the project by simply clicking on it
  e. Start acquisition to generate the full 2D tile scan image (Figure 8 → 16)
    ***Note:*** This may take less than 10 min per femur under current microscope settings.
32. Merge the tile images.
  a. Open the Project:
    i. Click the arrowhead to open the generated image file
    ii. Name the image
      **CRITICAL:** To generate a new image in the same project, select the project and click Start again. Alternatively, create a new project if the images belong to a different project.
  b. Open the Mosaic Merge function (Figure 8 → 17)
  c. Select the tile scanned image
  d. Select Smooth for Blend
  e. Merge
    ***Note:*** This merges the tiles and generates the 2D image of the entire femur.
    **CRITICAL:** We strongly recommend stitching the tile scan images directly from LAS X Navigator before dragging into Imaris. Using another stitching tool may cause image-content misalignment and unnecessary file size inflation.
33. Define regions of interest (ROIs) for 3D imaging
  a. Go back to Acquisition
  b. Click on the arrow (Figure 8 → 18)
  c. Select the tile scan rectangle
  d. Delete it (Figures 8 and 19)
  e. Double-click on the middle of the femur
  f. Zoom in with the mouse and start live
  g. Open the z-stack menu (Figure 8 → 20):
    i. Change the z-position by turning the focus knob in one direction
    ii. Click Set Begin to define the z-start position
    iii. Turn the focus knob in the other direction
    iv. Click Set End to define the z-end position
      ***Note:*** In this protocol, we defined the Set Begin and Set End positions so that the z-depth is ∼60 μm, at which all four signals (blue, green, red, and far-red) are clearly visible at both the lower and higher z-positions. This is because the green, red, and far-red channels can be detected to greater depths (up to ∼88 μm), whereas the blue channel signal tends to fade beyond ∼60 μm.
  h. Press Start to acquire.
    ***Note:*** This will generate a 3D reconstruction of the selected region in about 7 min. To image another ROI of the same bone (e.g., the femoral head), double-click the desired region, then press Start.
    **CRITICAL:** For quantification purposes, all the regions of interest (ROIs) must be imaged at the same z-depth, where all are clearly visible at both the lower and higher z-positions.

### Image analysis and segmentation in Imaris 10.2.0 (or higher)

**Timing: 30–40 min per femur**

Here, we describe the image analysis pipeline using Imaris 10.2.0 for maximum intensity projection, single cell detection by multiple signal intensity, surface segmentation of blood vessels and single cells of both 2D tile scan and 3D image of the femur.

34. Convert files using Imaris File Converter.
  a. Use Imaris File Converter version 9.5 (or plus) to convert the stack of.lif files resulting from the image acquisition into an.ims file.

***Note:*** This protocol generates relatively small image files, approximately 430 MB for the 2D tile scan slice and ∼180 MB for the 3D z-stack, making them easy to store, transfer, and analyze.

35. Import image files into Imaris.
  a. Open Imaris 10.2.0.
  b. Create a new folder by clicking on the new folder icon.
  c. Open the folder by double-clicking.
  d. Drag in the.ims file.
  e. Open the image by double-clicking:
    i. Show the Display Adjustment using Command+D (Mac) or Control+D (Windows).
    ii. Explore the image (2D or 3D) with the mouse.
    iii. Clicking through the channels.
    iv. Adjusting brightness and contrast if necessary.

**Figure 9:**
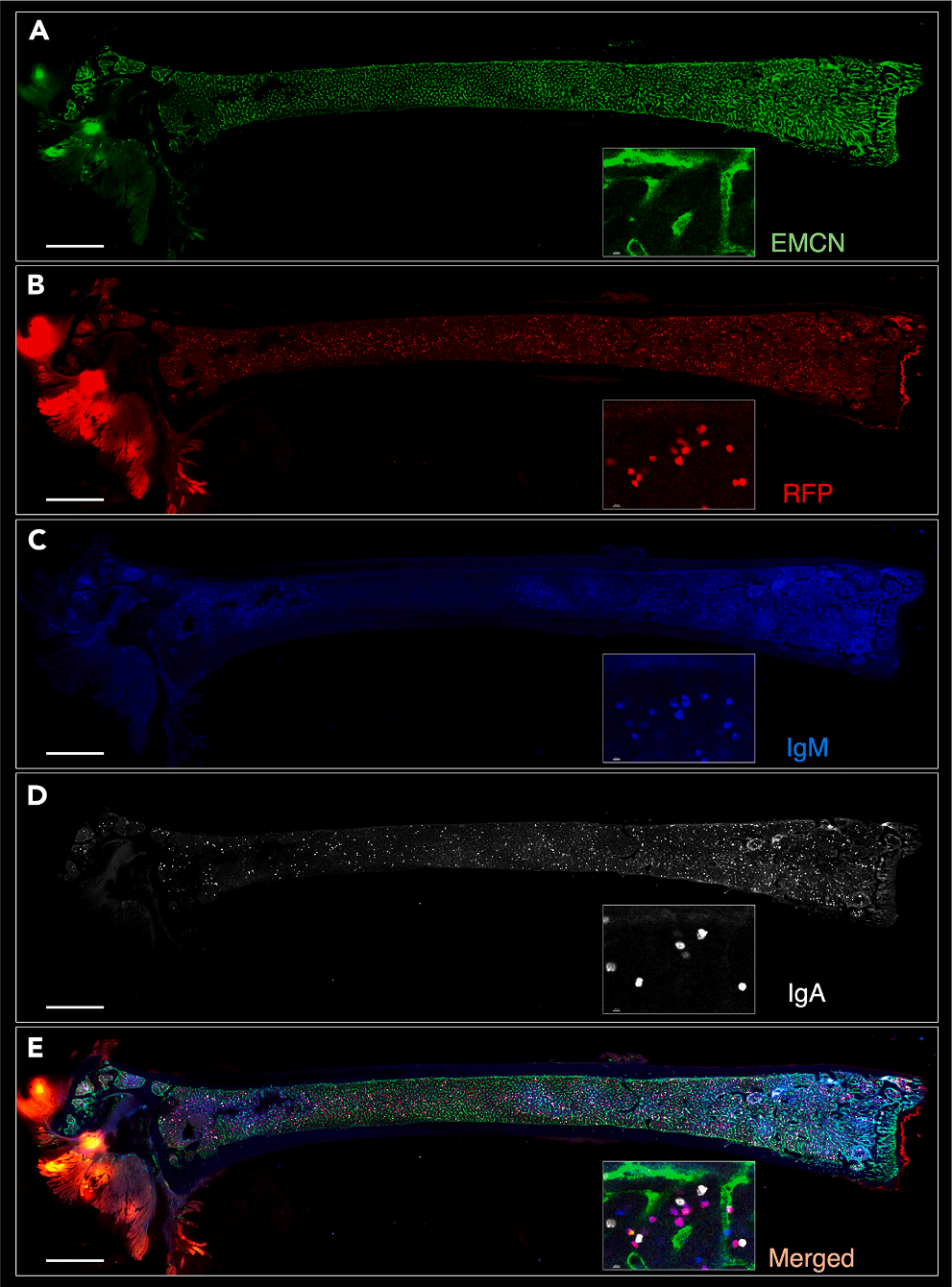
2D tile can visualization and maximum intensity projection (MIP) of multicolor-stained femur (A) Endomucin (EMCN) staining highlights the sinusoidal vessels throughout the femur. (B) RFP^+^ plasma cells (total timestamped) are distributed along the diaphysis and metaphyseal regions. (C) IgM^+^ plasma cells. (D) IgA^+^ plasma cells. (E) Merged image of all channels (EMCN in green, RFP in red, IgM in blue, IgA in white) across the entire femur. The insets show the higher-magnification views. Scale bar = 1000 μm.

**Figure 10:**
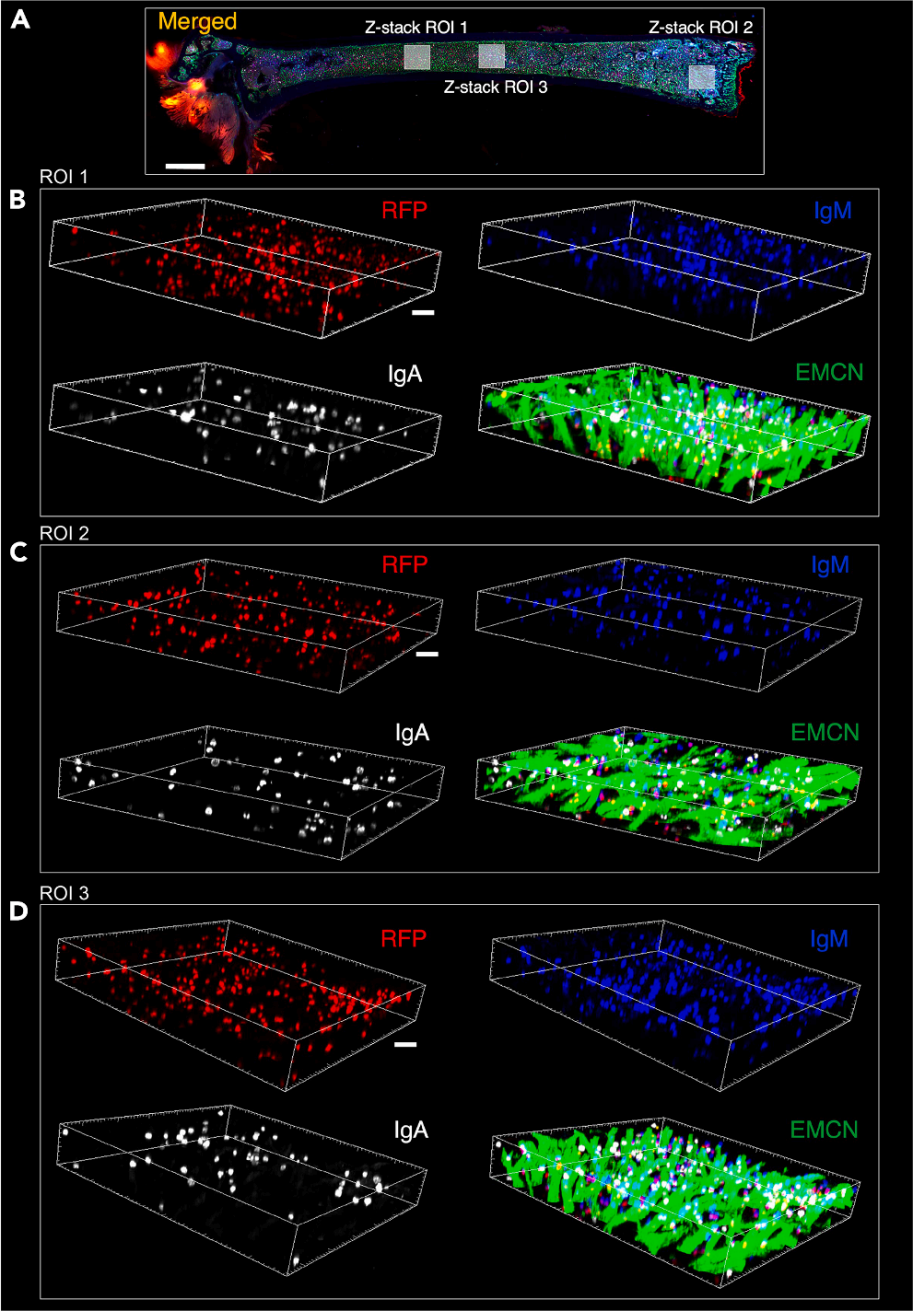
High-Resolution 3D z-stack Reconstructions of three regions of interest (ROIs) from the femur (A) Overview of the femur showing a 2D tile scan projection of the entire sample, with selected regions of interest (ROIs) indicated. (B–D) High-resolution 3D z-stack reconstructions of individual ROIs selected from the femur. Each ROI was defined by double-clicking on the region of interest in the tile scan. Individual channels are shown for RFP^+^ PCs (red), IgM^+^ PCs (blue), IgA^+^ PCs (white), and vasculature (EMCN, green). A target z-depth of ∼60 μm was set for each ROI; the final acquired z-stack, as measured by LAS X and Imaris, was ∼89 μm. z-stack images were acquired at a resolution of 512 × 512 × 89 (x, y, z). Voxel size was 1.14 × 1.14 × 1.00 μm. Scale bars: (A) 1000 μm; (B–D) 50 μm (Bottom panels).

***Note:*** The 2D tile scan and the 3D reconstructed volumes of the ROIs in this protocol are shown in Figures 9 and 10, following Display Adjustment for each channel and the maximum intensity projection (MIP) of the four channels (Blue/IgM = Channel 1, Gray/IgA = Channel 2, Green/Endomucin = Channel 3, Red/RFP = Channel 4).

### Create single IgM^+^ and double IgM^+^ RFP^+^ plasma cell spots from ROI 1

**Timing: ∼ 5 min per ROI**

**Figure 11:**
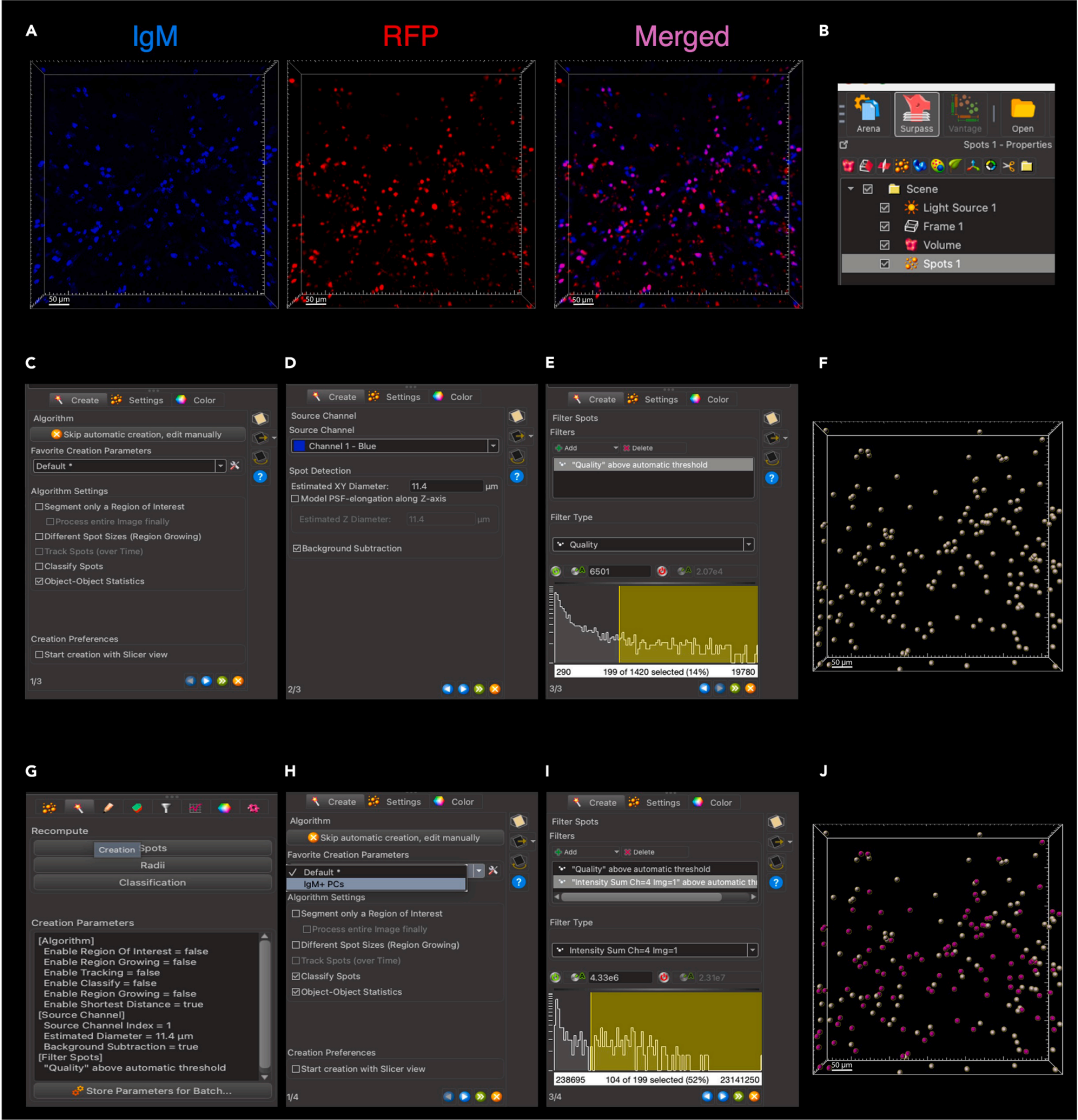
Identification strategies of total single IgM^+^ cells and double IgM^+^ RFP^+^ plasma cells of ROI 1 (A) Representative IgM (blue), RFP (red), and merged channels from ROI 1. (B and C) In Surpass, a new Spots object is created. In the Algorithm Settings, Object-Object Statistics is enabled, and Start creation with Slicer view is left unchecked. (D) Source Channel is set to Channel 1 (IgM). Estimated XY Diameter (11.4 μm) and Background Subtraction are confirmed as automatically determined by Imaris. (E and F) Quality-based filtering is applied to detect IgM^+^ PCs. The Quality slider is adjusted to exclude low-signal noise. Clicking Finish generates the Spots object representing all IgM^+^ cells. (G) Creation parameters are saved as a Favorite under the label IgM^+^ PCs for batch processing. (H) A second Spots object is created using the saved IgM^+^ PCs parameters. (I) An additional filter “Intensity Sum Ch=4 Img=1” is applied to select IgM^+^ PCs that also contain RFP signal. The slider is adjusted to remove weak signal and retain bright RFP^+^ IgM^+^ double-positive cells. (J) Final Spots representing double-positive (RFP^+^ IgM^+^) PCs are generated.

Here, we identify the total immunoglobulin M-expressing cells and then use the RFP signal intensity to identify the recombined IgM^+^ plasma cells (Figure 11A). We demonstrate this for one ROI of the femur using this protocol.

36. Open the image dataset.
  a. Launch Imaris and load the corresponding image file.
  b. Confirm that all channels are correctly assigned:
    i. Inspect the IgM channel (Alexa 405) and RFP channel (Alexa 555.
    ii. Ensure the signal-to-noise ratio is sufficient for segmentation.
37. Create Spots for IgM signal (Figure 11).
  a. In Surpass, add a new Spot by clicking the ‘Spots object’ icon.
  b. Select Object-object statistics in Algorithm Settings.
  c. Ensure Start creation with Slicer view is unchecked (Figures 11B and 11C).
  d. Click next (Blue arrowhead):
    i. Set Source Channel = Channel 1-Blue (Figure 11D).
    ii. Confirm the Estimated XY Diameter is 11.4 μm.
    iii. Ensure Background Subtraction is checked.
      ***Note:*** Imaris autofills these fields based on the image content
  e. Click next to apply the Quality threshold for IgM^+^ cells (Figure 11E)
  f. Identify the Filter Spots panel (Figure 11E):
    i. Adjust the Quality threshold by moving the slider right (to remove) or left (to include) low-signal background.
    ii. Keep the brightest IgM^+^ candidate cells.
  g. Click Finish (green arrowhead) to create the Spots object representing all IgM^+^ cells (Figure 11F).
  h. Click on the Creation icon (Figure 11G).
  i. Store Parameters for Batch in the Favorite Creation Parameters as IgM^+^ PCs.
    ***Note:*** The Imaris-prompted threshold values shown in this protocol depend on imaging conditions (e.g., microscope configuration, acquisition settings, and staining).
    **CRITICAL:** We advise users to analyze the first image, set the initial threshold values (Imaris-defined or user-defined), and apply them across multiple image datasets to ensure consistency in comparative analyses.
38. Identify IgM^+^ RFP^+^ double positive cells.
  a. Add a new Spot (Figure 11H):
    i. Select IgM^+^ PCs in the Favorite Creation Parameters.
    ii. Click next.
  b. Click on the + sign to add a filter (Figure 11I).
  c. Select filter type “Intensity Sum Ch=4 Img =1” above automatic threshold:
    i. Adjust the filter by moving the slider right (to remove) or left (to include) low-signal background.
    ii. Keep the brightest IgM^+^ RFP^+^ candidate cells.
  d. Click Finish to create the final set of double-positive Spots (Figure 11J).

***Note:*** This selects the IgM^+^ cells with RFP signal Intensity in Channel 4.

### Create single IgA^+^ and double IgA^+^ RFP^+^ plasma cell spots from ROI 1

**Timing: ∼ 5 min per ROI**

**Figure 12:**
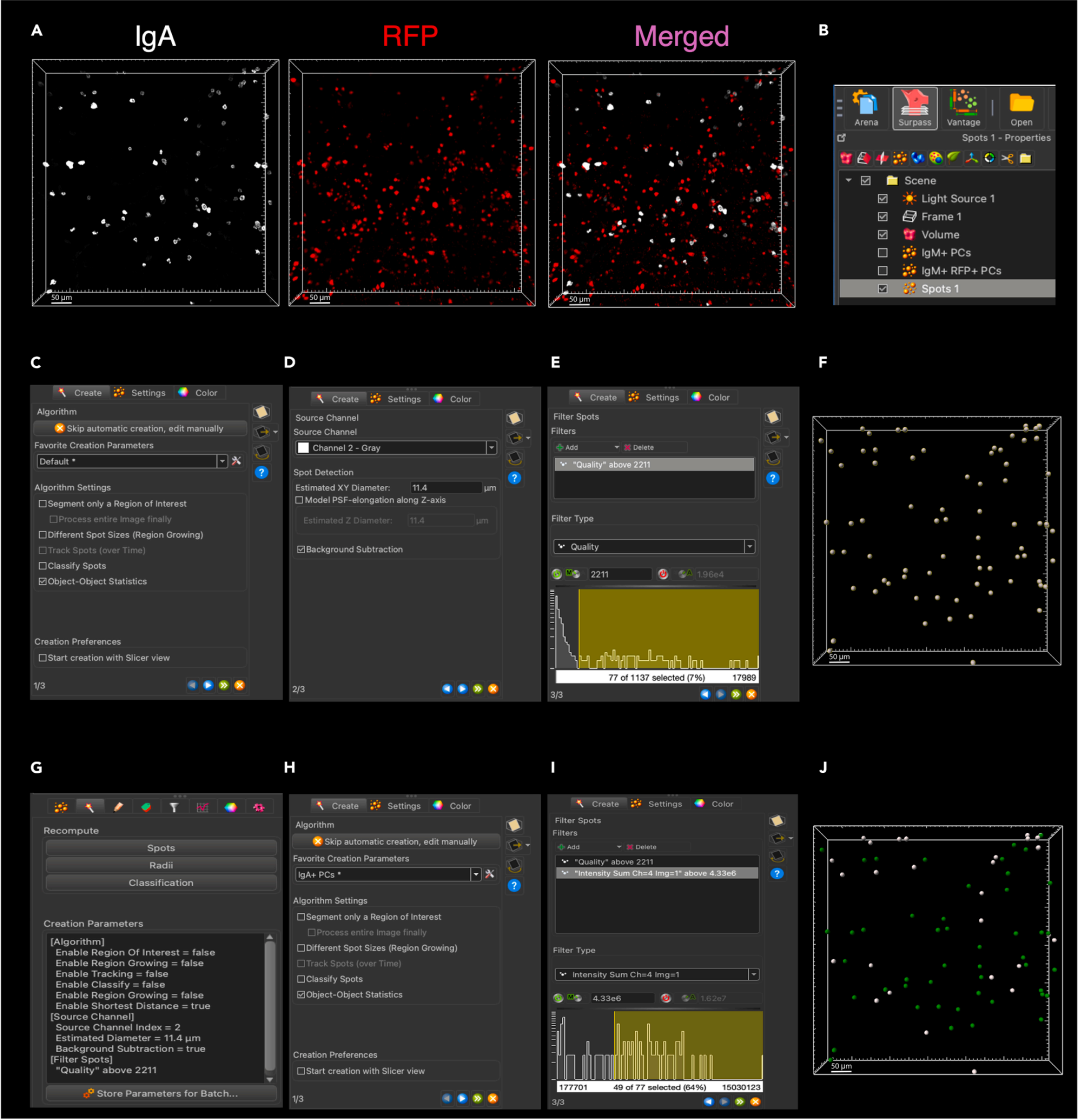
Identification strategies of total single IgA^+^ cells and double IgA^+^ RFP^+^ plasma cells of ROI 1 (A) Representative IgA (white), RFP (red), and merged views from ROI 1. Scale bars = 50 μm. (B and C) In Surpass, a new Spots object is created. In the Algorithm Settings, Object-Object Statistics is enabled, and Start creation with Slicer view is left unchecked. (D) Source Channel is set to Channel 2 (IgA). Estimated XY Diameter (11.4 μm) and Background Subtraction are confirmed as automatically determined by Imaris. (E and F) A Quality-based filter is applied to detect IgA^+^ cells. The Quality slider is adjusted to exclude low-intensity background. Clicking Finish generates the Spots object representing all IgA^+^ cells. (G) Creation parameters are saved in Favorite Creation Parameters under the label IgA^+^ PCs for batch analysis. (H) A second Spots object is created using the saved IgA^+^ PCs parameters. (I) An additional filter “Intensity Sum Ch=4 Img=1” is added to select IgA^+^ PCs that also contain RFP signal. The slider is adjusted to remove weak RFP background and retain bright RFP^+^ IgA^+^ cells. (J) Final Spots representing double-positive (IgA^+^RFP^+^) PCs are generated.

Here, we identify the total immunoglobulin A-expressing cells and then use the RFP signal intensity to identify the recombined IgA^+^ plasma cells (Figure 12A) within the same ROI.

39. Create Spots for IgA signal (Figure 12).
  a. Add a new Spot by clicking the ‘Spots object’ icon.
  b. Select Object-object statistics in Algorithm Settings.
  c. Ensure Start creation with Slicer view is unchecked (Figure 12C).
  d. Click next (Blue arrowhead):
    i. Set Source Channel = Channel 2-Grey (Figure 12D).
    ii. Confirm the Estimated XY Diameter is 11.4 μm.
    iii. Ensure Background Subtraction is checked.
  e. Click next to apply the Quality threshold for IgA^+^ cells (Figure 12E).
  f. From the Filter Spots panel (Figure 12E):
    i. Adjust the Quality threshold by moving the slider right (to remove) or left (to include) low-signal background.
    ii. Keep the brightest IgA^+^ candidate cells.
  g. Click Finish (green arrowhead) to create the Spots object representing all IgA^+^ cells (Figure 12F).
  h. Click on the Creation icon (Figure 12G).
  i. Store Parameters for Batch in the Favorite Creation Parameters as IgA^+^ PCs.
40. Identify IgA^+^ RFP^+^ double positive cells.
  a. Add a new Spot:
    i. Select IgA^+^ PCs in the Favorite Creation Parameters ((Figure 12H).
    ii. Click next.
  b. Click on the + sign to add a filter (Figure 12I).
  c. Select filter type “Intensity Sum Ch=4 Img =1” above automatic threshold:
    i. Set the lower intensity threshold to 4.33e6.
      ***Note:*** 4.33e6 is the same value as that prompted for the RFP signal in IgM^+^ cells
  d. Click Finish to create IgA^+^ RFP^+^ double-positive Spots (Figure 12J).

### Create single RFP^+^ and double IgM^-^ IgA^-^ RFP^+^ plasma cell spots from ROI 1

**Timing: ∼ 10 min per ROI**

This section describes how to identify total RFP^+^ plasma cells and RFP^+^ IgM^-^ IgA^-^ plasma cell subsets within the same ROI using the Spot tool.

**Figure 13:**
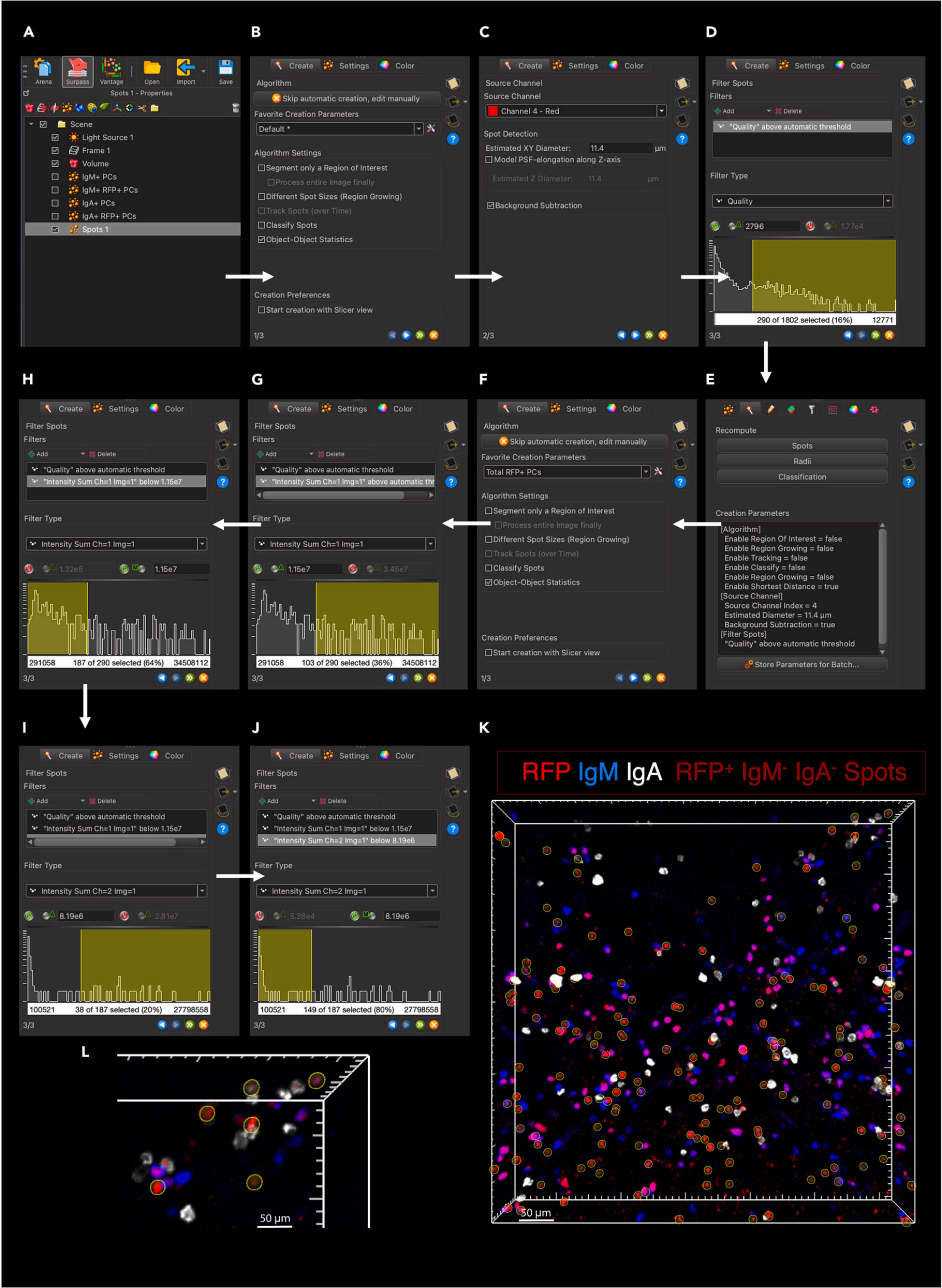
Identification strategies of single RFP^+^ and double RFP^+^ IgM^-^ IgA^-^ plasma cells of ROI 1 (A) In Surpass, a new Spots object is created. In the Algorithm Settings, Object-Object Statistics is selected, and Start creation with Slicer view is left unchecked. (B and C) Source Channel is set to Channel 4 (RFP). Estimated XY Diameter (11.4μ m) and Background Subtraction are confirmed as automatically determined by Imaris. (D) A Quality threshold is applied to detect bright RFP^+^ PCs. The Quality slider is adjusted to exclude low-signal background and retain high-intensity cells. Clicking Finish generates the **initial Spots** object representing all RFP^+^ PCs. (E) Creation parameters for RFP^+^ PCs are saved under the label Total RFP^+^ PCs for batch analysis. (F and G) A second Spots object is generated using Total RFP^+^ PCs parameters. An additional filter “Intensity Sum Ch=1 Img=1” is added to assess IgM intensity within RFP^+^ cells. (H and I) To exclude IgM^+^ cells, the Lower Threshold value from the filter is copied, the Lower Threshold is deactivated, and the copied value is pasted into the Upper Threshold. This reverses the filter logic, removing all RFP^+^ cells with IgM signal above the cutoff and retaining only IgM^-^ RFP^+^ PCs. (I and J) A second filter “Intensity Sum Ch=2 Img=1” is added to remove IgA^+^ cells. Again, the Lower Threshold value is copied, the Lower Threshold is deactivated, and the value is pasted into the Upper Threshold to exclude high IgA signal. Applying this filter yields the final RFP^+^ IgM^+^ IgA^+^ PC population. (K) Final visualization of segmented RFP^+^ IgM^-^ IgA^-^ PC within the 3D volume, shown alongside IgM^+^, IgA^+^, and double-negative populations for reference. (L) Inset illustrating exclusion of IgM^+^ and IgA^+^ cells from the RFP^+^ population.
    iii. Click next (Blue arrowhead).
  b. Set Source Channel = Channel 4-Red (Figure 13C):
    i. Confirm the Estimated XY Diameter is 11.4 μm.
    ii. Check Background Subtraction.
  c. Click next to apply the Quality threshold for RFP^+^ plasma cells (Figure 13D):
    i. Identify the Filter Spots panel.
    ii. Adjust the Quality threshold by moving the slider right (to remove) or left (to include) low-signal background.
    iii. Keep the brightest RFP^+^ candidate cells.
  d. Click Finish (green arrowhead) to create the Spots object representing all RFP^+^ plasma cells.
  e. Click on the Creation icon:
    i. Store Parameters for Batch in the Favorite Creation Parameters as Total RFP^+^ PCs (Figure 13E).

41. Create Spots for RFP^+^ plasma cells.
  a. Add spot using Spots creation wizard:
    i. Select Object-object statistics in Algorithm Settings.
    ii. Ensure Start creation with Slicer view is unchecked (Figures 13A and 13B).

**CRITICAL:** Apply the same threshold across all ROIs and samples for comparative analysis.

42. Exclude IgM^+^ IgA^+^ cells.
  a. Add a new Spot:
    i. Select Total RFP^+^ PCs in the Favorite Creation Parameters (Figure 13F)
    ii. Click next.
  b. Click the + sign to add a filter (Figure 13G)
  c. Select the filter type “Intensity Sum Ch=1 Img =1” above the automatic threshold:
    i. Copy the value in the Lower threshold box.
    ii. Click the green power button to deactivate the lower-threshold box (Figure 13G).
    iii. Activate the Upper threshold box by clicking on the red power button.
    iv. Paste that value in the Upper threshold box (Figure 13H).
    v. Click next to select RFP^+^ IgM^-^ plasma cells.
      ***Note:*** The Lower Threshold in the “Intensity Sum = Ch1 Img = 1” filter includes all RFP^+^ objects that are IgM^+^ above the selected value. By copying this value into the Upper Threshold, deactivating the Lower Threshold, and activating the Upper Threshold, Imaris then excludes all RFP^+^ objects with IgM intensity above that cutoff, thereby selecting only the IgM-negative population (RFP^+^ IgM^-^ PCs).
  d. Click on the + sign again to add a second filter.
  e. Select the filter type “Intensity Sum Ch=2 Img =1” above the automatic threshold (Figure 13I):
    i. Copy the value in the Lower threshold box.
    ii. Click on the green power button to deactivate the lower threshold box.
    iii. Activate the Upper threshold box by clicking on the red power button.
    iv. Paste that value in the Upper threshold box (Figure 13J).
  f. Click next to select RFP^+^ IgM^-^ IgA^-^ plasma cells, shown as brown spots in Figures 13K and 13L (close-up).
    ***Note:*** The Lower Threshold in the “Intensity Sum = Ch2 Img = 1” filter includes all RFP^+^ IgM^-^ objects that are IgA^+^ above the selected value. By copying this value to the Upper Threshold, deactivating the Lower Threshold, and activating the Upper Threshold, Imaris then excludes all RFP^+^ IgM^-^ objects with IgA intensity above that cutoff, thereby selecting only the IgA-negative population (RFP^+^ IgM^-^ IgA^-^PCs).

### Create IgM^+^ RFP^+^ plasma cell surface from ROI 1

**Timing: ∼ 5 min per ROI**

This section explains how to generate surface objects to identify IgM^+^RFP^+^ plasma cells within ROI 1 using intensity-based segmentation and channel filtering.

43. Create surface objects for IgM^+^ cells.
  a. Add a New Surface by clicking the ‘Surface Object’ icon (Figure 14A):
    i. Enable Object-object statistics.
    ii. Leave the Leave Start creation with Slicer view unticked.
    iii. Click next.

**Figure 14:**
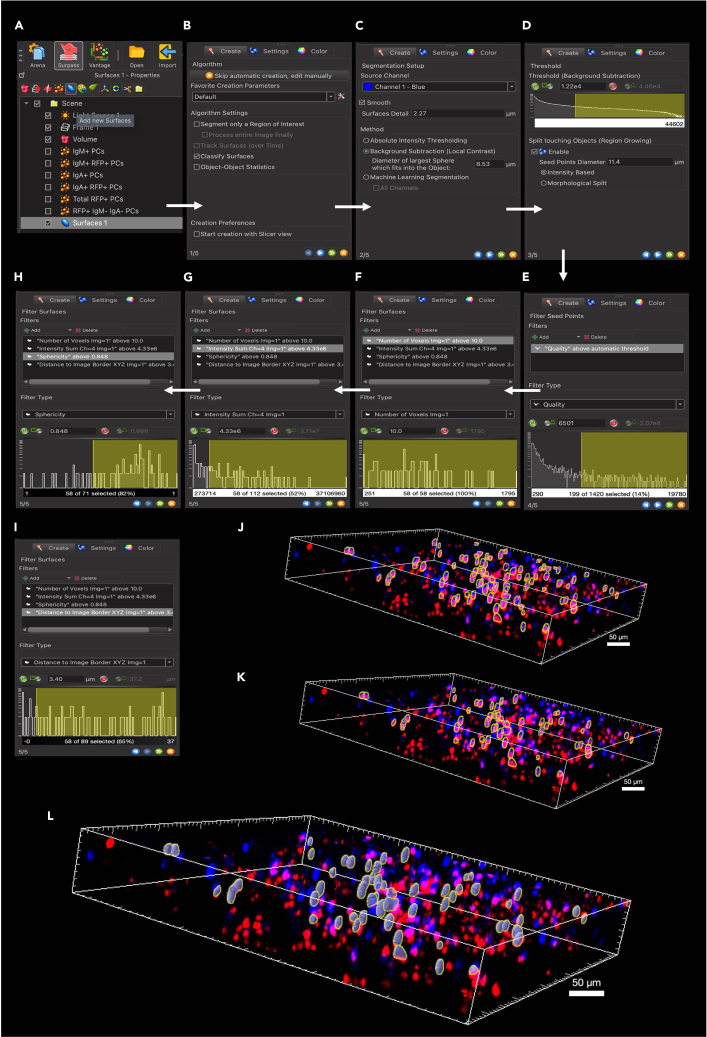
Surface segmentation of IgM^+^ RFP^+^ plasma cells of ROI 1 (A) In Surpass, add a new Surface object by clicking the Surface icon. (B) In the Algorithm panel, enable Object-Object Statistics, leave Start creation with Slicer view unchecked, and click Next. (C) Segmentation Setup: (i) Set Source Channel = Channel 1 (Blue, IgM); (ii) Enable Smoothing (default value); (iii) Enable Background Subtraction (keeping the default Diameter of the Largest Sphere). (D) Threshold (Background Subtraction): (i) Move the threshold slider to the right to eliminate the background; (ii) Enable Split Touching Objects; (iii) Keep the default Seed Points Diameter, (iv) Enable Intensity-Based Split and click Next. (E) Filter Seed Points: retain high-quality detections using “Quality above automatic threshold”. (F–I) Add the following filters: (i) “Number of Voxels Img=1”; (ii) “Intensity Sum Ch=4 (RFP)”; (iii) “Sphericity”; and “Distance to Image Border XYZ Img=1”. (F) Set Number of Voxels Img=1 > 10 (more than 10 voxels). (G) For Intensity Sum Ch=4 (RFP), set the lower threshold to 4.33e6. (H) Apply the Sphericity filter to exclude dividing cells/doublets. (I) Apply Distance to Image Border XYZ Img=1. (J) Detecting IgM^+^ RFP^+^ PC surfaces before the Distance to Image Border XYZ filter is applied. Many cells are truncated at volume edges when the lower threshold = 0 μm. (K) Excluding all truncated cells to retain only complete cells when the lower threshold is set to 3.40 μm. (L) Final 3D visualization of segmented IgM^+^ RFP^+^ PC in ROI 1.
  b. Do the following under the Segmentation Setup (Figure 14C):
    i. Select Channel 1-Blue in Source Channel.
    ii. Enable Smoothing.
    iii. Leave the set default value.
  c. Check Background Subtraction in Method:
    i. Leave the set default value of Diameter of Largest Sphere
    ii. Click next.
      ***Note:*** The surface segmentation algorithm automatically defines a value for the diameter of the largest sphere that fits into the 3D Object (IgM^+^ cell).
      **CRITICAL:** This value ensures correct seed placement within IgM^+^ objects, allowing region growing towards the actual cell boundaries and enabling accurate detection of individual cells even when they are adjacent.
  d. Under Threshold (Background Subtraction, Figure 14D), move the slider to the right until background signals are excluded from detection:
    i. Enable Split touching Objects.
    ii. Leave the Seed Points Diameter value as prompted (default).
    iii. Enable Intensity-Based Split
    iv. Click next to Figure 14E
      ***Note:*** Intensity-based splitting uses local intensity maxima to separate individual cells, allowing accurate segmentation under intensity-driven region growing, even when cells touch or have irregular shapes.
44. Identify 3D IgM^+^ cells with RFP signal
  a. Add the following filters:
    i. “Intensity Sum Ch= 4.
    ii. “Sphericity”.
    iii. “Distance to Image Border XYZ (Img=1)”.
  b. Click on filter Number of Voxels Img= 1.
  c. Set its lower threshold value to 10.
    ***Note:*** Here, we applied “Number of Voxels Img=1” > 10 as a conservative cutoff to exclude only tiny, segmented objects (artefacts), which typically occupy fewer than 10 voxels. In contrast, true cells occupy orders of magnitude more.
  d. Click on filter “Intensity Sum Ch= 4”.
  e. Set its lower threshold to 4.33e6.
    ***Note:*** 4.33e6 is the same lower threshold value used to detect IgM^+^ RFP^+^ Spots (Figure 11).
  f. Click “Sphericity” to exclude merged cells (Figure 14H).
    ***Note:*** Sphericity filtering removes merged cells (doublets or aggregates), as these objects deviate from spherical geometry and exhibit elongated or irregular shapes with lower sphericity values.
    **CRITICAL:** Because cellular density and spatial interactions vary across samples and within different regions of the same tissue, fixed sphericity thresholds may not accurately capture true single-cell morphology. Therefore, thresholds were determined using the Imaris automatic function, which adapts to dataset-specific object characteristics. Users should visually validate segmentation to ensure accurate exclusion of merged cells. For comparative analyses, consistent filtering criteria should be applied across datasets acquired under similar conditions.
  g. Click the filter “Distance to Image Border XYZ (Img=1)”:
    i. Explore the 3D image from different angles.
    ii. Adjust the lower threshold so that only fully captured single IgM^+^ RFP^+^ 3D objects within the 3D volume are included.
      ***Note:*** With a lower threshold value of 3.40 μm, we have excluded objects cut by the image boundaries. We illustrated this in Figure 14J (lower threshold = 0 μm, before filtering), and Figure 14K (lower threshold = 3.40 μm, after filtering). Figure 14L is a magnification of Figure 14K for a better appreciation of the 3D objects within the volume.
      **CRITICAL:** This step is essential because partially captured cells produce incomplete segmentation and should therefore be excluded to ensure reliable quantification.

### Create IgA^+^ RFP^+^ plasma cell surface from ROI 1

**Timing: ∼ 5 min per ROI**

As for IgM^+^ RFP^+^ plasma cells, this section uses the same strategy to generate IgA^+^ RFP^+^ surface objects within the same ROI.

**Figure 15:**
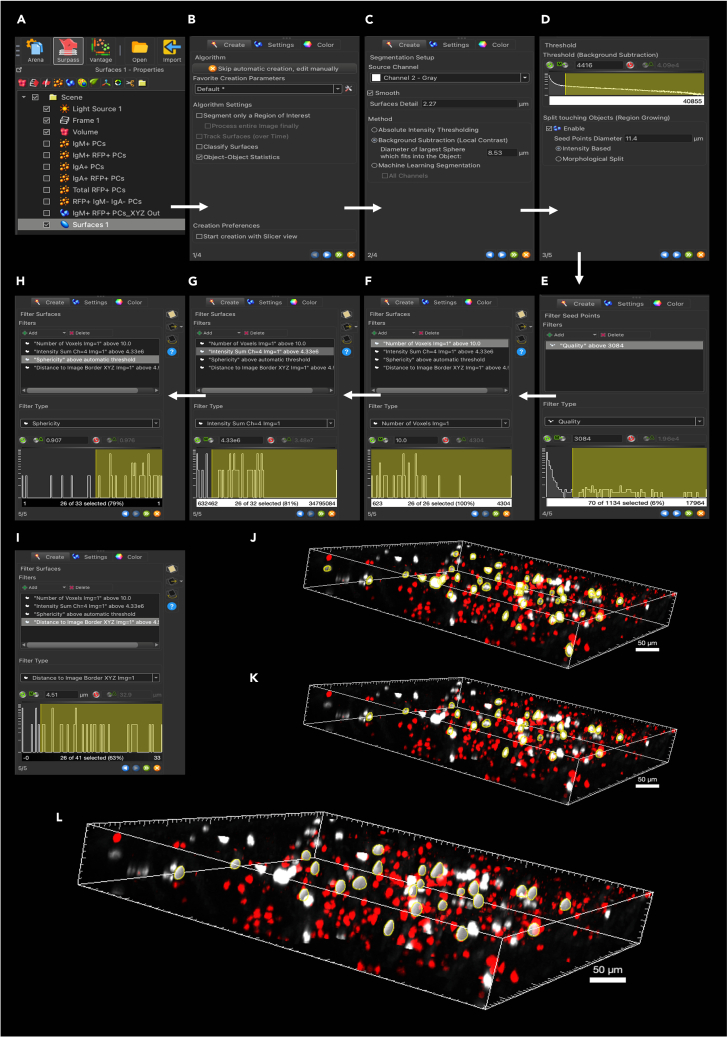
Surface segmentation of IgA^+^ RFP^+^ plasma cells of ROI 1 (A) In Surpass, add a new Surface object by clicking the Surface icon. (B) In the Algorithm panel, enable Object-Object Statistics, leave Start creation with Slicer view unchecked, and click Next. (C) Segmentation Setup: (i) Set Source Channel = Channel 2 (Gray, IgA); (ii) Enable Smoothing (default value); (iii) Enable Background Subtraction (keeping the default Diameter of the Largest Sphere). (D) Threshold (Background Subtraction): (i) Move the threshold slider to the right to eliminate the background; (ii) Enable Split Touching Objects; (iii) Keep the default Seed Points Diameter, (iv) Enable Intensity-Based Split and click Next. (E) Filter Seed Points: retain high-quality detections using “Quality above automatic threshold”. (F–I) Add the following filters: (i) “Number of Voxels Img=1”; (ii) “Intensity Sum Ch=4 (RFP)”; (iii) “Sphericity”; and “Distance to Image Border XYZ Img=1”. (F) Set Number of Voxels Img=1 to 10. (G) For Intensity Sum Ch=4 (RFP), set the lower threshold toe6. (H) Apply the Sphericity filter to exclude dividing cells/doublets. (I) Apply Distance to Image Border XYZ Img=1. (J) Detecting IgA^+^ RFP^+^ PC surfaces before the Distance to Image Border XYZ filter is applied. Many cells are truncated at volume edges when the lower threshold = 0 μm. (K) Excluding all truncated cells to retain only complete cells when the lower threshold is set to 4.51 μm. (L) Final 3D visualization of segmented IgA^+^ RFP^+^ PCs in ROI 1.
  b. Do the following under the Segmentation Setup (Figure 15C):
    i. Select Channel 2-Gray in Source Channel.
    ii. Enable Smoothing.
    iii. Leave the set default value.
  c. Check Background Subtraction in Method:
    i. Leave the set default value of Diameter of Largest Sphere.
    ii. Click next.
  d. Under Threshold (Background Subtraction, Figure 15D), move the slider to the right until background signals are excluded from detection:
    i. Enable Split touching Objects.
    ii. Leave the Seed Points Diameter value as prompted (default).
    iii. Enable Intensity-Based Split.
    iv. Click next to Figure 15E.
46. Identify 3D IgA^+^ cells with RFP signal.
  a. Add the following filters:
    i. “Intensity Sum Ch= 4.
    ii. “Sphericity”.
    iii. “Distance to Image Border XYZ (Img=1)”.
  b. Click on filter Number of Voxels Img= 1.
  c. Set its lower threshold value to 10.
  d. Click on filter “Intensity Sum Ch= 4”.
  e. Set its lower threshold to 4.33e6.
  f. Click “Sphericity” to exclude merged cells (Figure 15H).
  g. Click the filter “Distance to Image Border XYZ (Img=1)”:
    i. Explore the 3D image from different angles.
    ii. Adjust the lower threshold so that only fully captured single IgA^+^ RFP^+^ 3D objects within the 3D volume are included.

***Note:*** With a lower threshold of 4.51 μm, we have excluded objects that are cut by the image boundaries. We illustrated this in Figure 15J (lower threshold = 0 μm, before filtering) and Figure 15K (lower threshold = 4.51 μm, after filtering). Figure 15L is a magnification of Figure 15K to better appreciate the 3D objects within the volume.

45. Create surface objects for IgA^+^ cells.
  a. Add a New Surface by clicking the ‘Surface Object’ icon (Figure 15A):
    i. Enable Object-object statistics.
    ii. Leave the Leave Start creation with Slicer view unticked.
    iii. Click next.

### Image analysis: Statistics extraction

**Timing: 10–20 min per ROI**

Here, we describe how to read and/or export statistics for plasma cell Spots and segmented 3D Surfaces.

47. Extract the population-level measurements.
  ***Note:*** We exemplified IgM^+^ cell Spots and Surfaces in Figure 16. However, the procedure is the same for all other populations.
  a. Select the segmented object of interest (IgM^+^ cell Spots):
    i. Click the Statistics icon.
    ii. Read the Total Number of Spots (Figure 16A).
  b. Click Export All Statistics to File at the very bottom.
  c. Save the CSV files to a designated folder.
  d. Select IgM^+^ RFP^+^ PCs XYZ Out Surface:
    i. Click the Statistics icon.
    ii. Read the Total Number of Disconnected Components/Total Number of Surfaces (Figure 16B).
  e. Export All Statistics to File.
  f. Save the CSV files.

**Figure 16:**
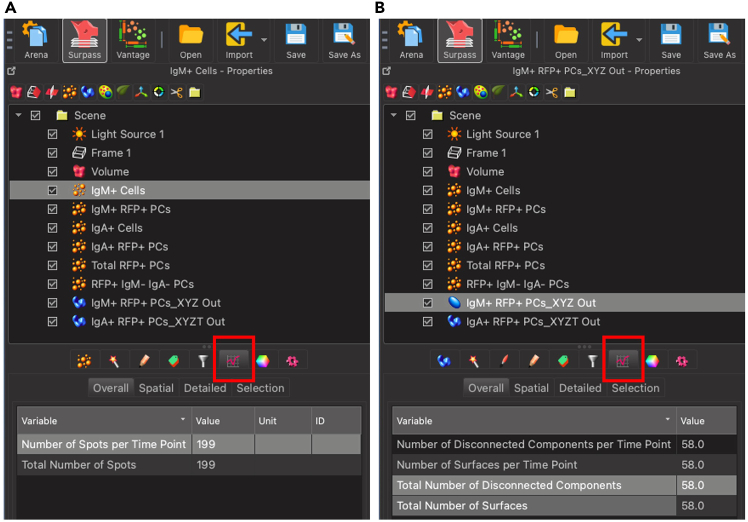
Extraction of quantitative statistics for Spots and Surfaces (A) To obtain the total number of IgM^+^ cells, select the IgM^+^ Cells (Spots) and click the Statistics icon (highlighted). Under the Selection tab, read the value for Total Number of Spots and Export all statistics by clicking. Export All Statistics to File (see the very bottom not shown here) and save the CSV files in a user-defined folder. (B) Select the IgM^+^RFP^+^PCs_XYZ Out (Surfaces) object and click the Statistics icon. Under the Selection tab, read Total Number of Disconnected Components (Total Number of Surfaces). Export the statistics as above.
48. Perform downstream analysis.

The exported Statistics are then analysed using GraphPad Prism or equivalent software.

***Note:*** We assume that the experimenter has already mastered GraphPad Prism. Tutorials on how to use this software are available on YouTube.

## Expected outcomes

Export All Statistics to File under Imaris 10.2.0 generates 89 quantitative CSV files for Spot-detected cells and 71 quantitative CSV files for Surface-segmented cells. The experimenter can choose the files relevant to the research questions. Here, we analyzed the cell numbers from the Overall.csv file and cell morphology from Volume.csv and Sphericity.csv files.

## Quantification and statistical analysis

Imaris exports per-object statistics as.csv files. For each bone, quantify plasma cell subsets as total Spots or Surfaces within each imaged region of interest (ROI). For morphology, use per-cell Volume and Sphericity outputs from the corresponding.csv tables. Define inclusion criteria before analysis (e.g., exclude objects truncated at volume edges using the ‘Distance to Image Border’ filter described here, and exclude small artefacts using a conservative voxel cutoff). Summarize data per bone (e.g., median per-cell morphology per ROI, or pooled per-cell distributions) and analyze in GraphPad Prism. When comparing conditions, use biological replicates as the unit of replication (mice) and report the number of bones and ROIs analyzed. Statistical tests depend on distribution; non-parametric tests are appropriate for per-mouse summary values.

**Figure 17:**
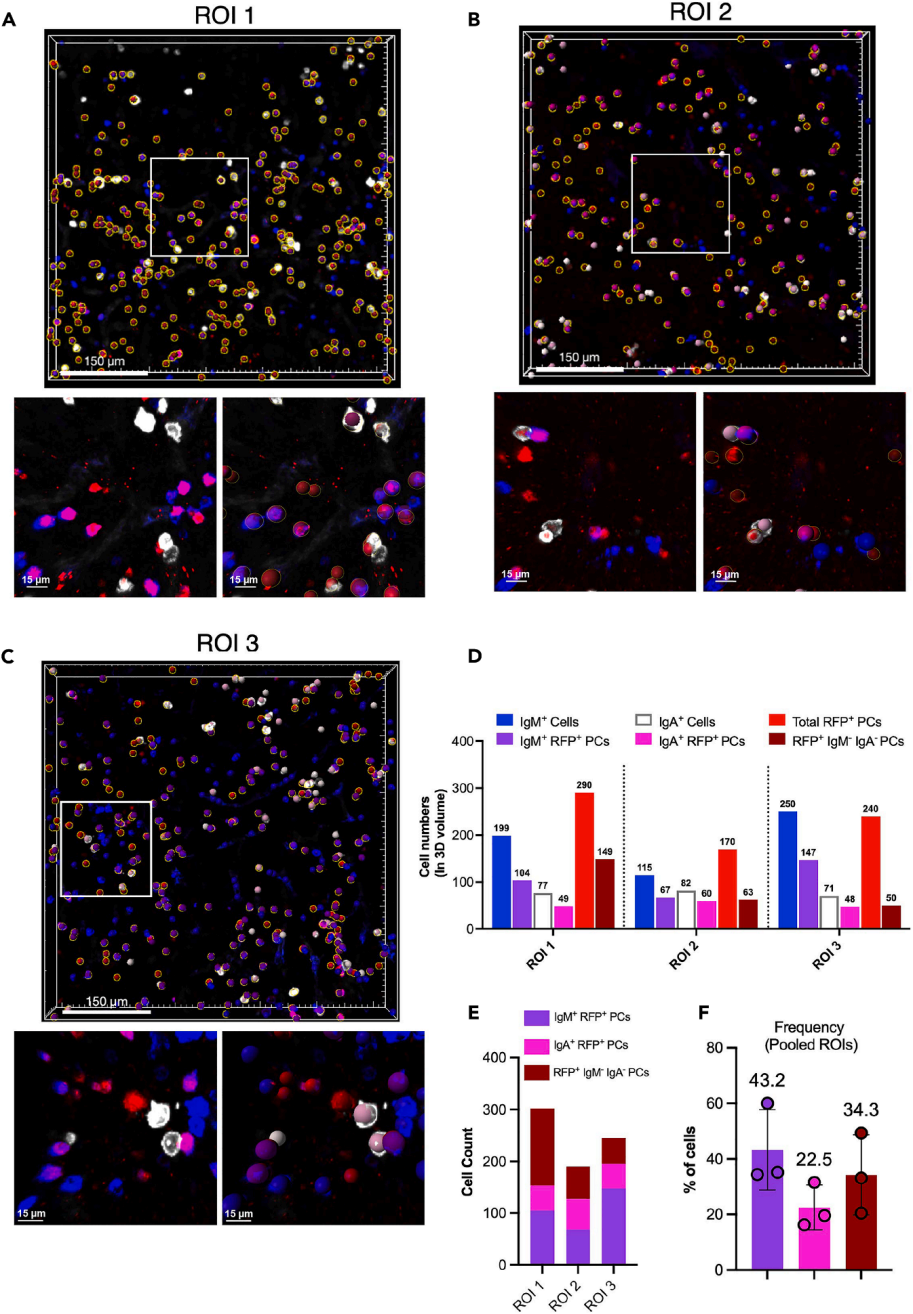
Quantification of segmented plasma cell subsets in three ROIs from the 3D volume (A–C) 3D renderings of the three ROIs showing segmented IgM^+^Spots (blue), IgM^+^ RFP^+^ Spots (purple), IgA^+^ Spots (gray/white), IgA^+^ RFP^+^ Spots (pink), RFP^+^ Spots (red), and RFP^+^IgM^-^IgA^-^ Spots (brown). Yellow surface outlines highlight the total RFP^+^ cell Spots. White boxes mark zoomed areas shown below each ROI. Insets show high-resolution examples of individual cell subsets. (D–F) Quantification of PC subsets in the three ROIs. Bar graphs summarizing cell numbers (D-E) extracted from Imaris statistics for each ROI. The mean frequency of each isotype across the three ROIs is presented in Graph F. Data illustrate regional variation in PC density and composition within the same femur. Error bars: standard deviation.

**Figure 18:**
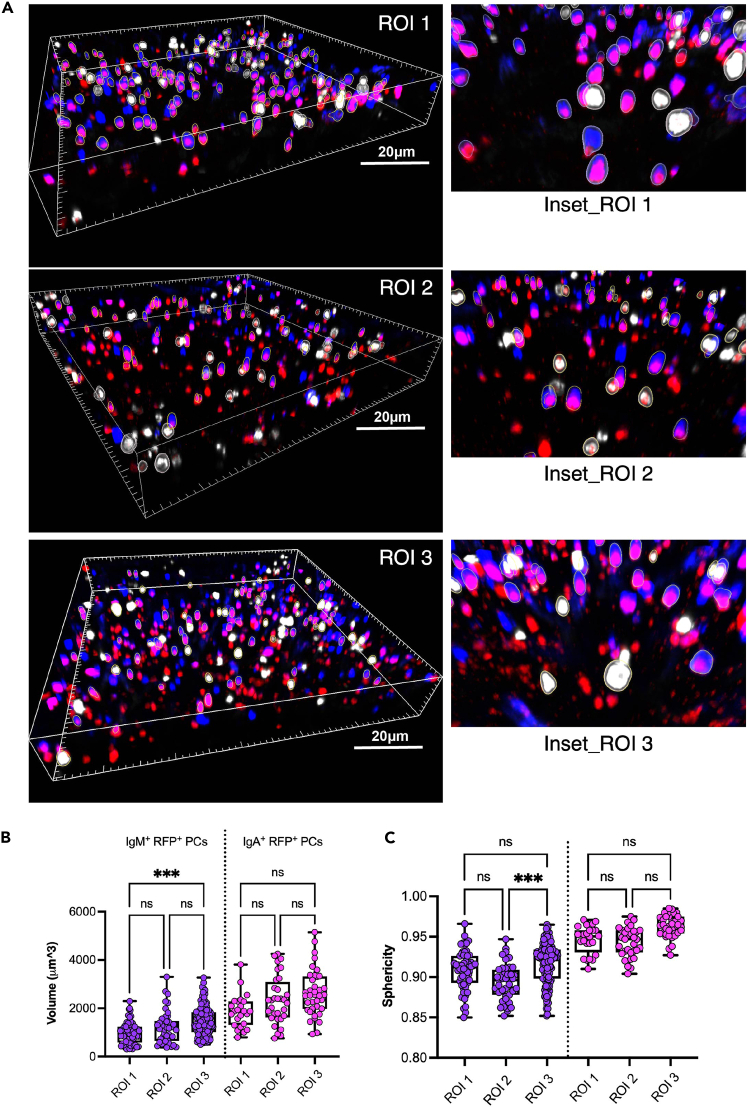
Morphological analysis of segmented plasma cell subsets in three ROIs from the 3D volume (A) 3D-rendered views of three ROIs, showing surface-segmented PCs: IgM^+^ (blue), IgA^+^ (white), and RFP^+^ (red). Double-positive IgM^+^RFP^+^ and IgA^+^RFP^+^ PCs are outlined in yellow. Each ROI includes an inset for high-magnification viewing of the outlines. (B and C) Quantitative comparison of plasma cell subset morphology across the three ROIs. Scatterplots show the distributions and medians of cell volume and sphericity for the IgM^+^RFP^+^ and IgA^+^RFP^+^ plasma cell subsets. Data were extracted from the Imaris Volume.csv and Sphericity.csv files. Statistical analyses were performed using a non-parametric multiple-comparisons ANOVA test. ns: non-significant; ∗∗∗*p* < 0.0001.

***Note:*** All steps shown in Figures 11, 12, 13, 14, 15, and 16, performed on ROI 1, were also applied to ROI 2 and ROI 3 of the same bone. The 3D renderings of the ROIs are shown in Figures 10, 17, and 18 (see Methods videos S1, S2, and S3 for representative 3D visualisations). Their comparative statistics are presented as histograms in Figures 17D, 17E, 18B, and 18C.

Methods video S1. ROI 1 The video display 3D animation views of the maximum intensity projections of Endomucin (Green), Red Fluorescence Protein (RFP), IgM (Blue) and IgA (White), IgM^+^ RFP^+^ PCs (Magenta Spots), IgA^+^ RFP^+^ PCs (Green Spots) and RFP^+^ IgM^-^ IgA^-^ PCs (Brown Spots). Image size: 512 μm x 512 μm x 89 μm (x,y,z). Voxel size: 1.14 x 1.14 x 1.00 [μm]. File size: 131.9 MB.

Methods video S2. ROI 2 The video display 3D animation views of the maximum intensity projections of Endomucin (Green), Red Fluorescence Protein (RFP), IgM (Blue) and IgA (White), IgM^+^ RFP^+^ PCs (Magenta Spots), IgA^+^ RFP^+^ PCs (Green Spots) and RFP^+^ IgM^-^ IgA^-^ PCs (Brown Spots). Image size: 512 μm x 512 μm x 89 μm (x,y,z). Voxel size: 1.14 x 1.14 x 1.00 [μm]. File size: 82.3 MB.

Methods video S3. ROI 3 The video display 3D animation views of the maximum intensity projections of Endomucin (Green), Red Fluorescence Protein (RFP), IgM (Blue) and IgA (White), IgM^+^ RFP^+^ PCs (Magenta Spots), IgA^+^ RFP^+^ PCs (Green Spots) and RFP^+^ IgM^-^ IgA^-^ PCs (Brown Spots). Image size: 512 μm x 512 μm x 89 μm (x,y,z). Voxel size: 1.14 x 1.14 x 1.00 [μm]. File size: 103.3 MB.

## Limitations

This protocol requires careful manual opening of the femur, which may introduce operator-dependent variability in the opening efficiency, antibody penetration and imaging depth. The algorithms for effective segmentation are already integrated into Imaris, which avoids the user relying on computational methods. However, segmentation may require dataset-specific adjustment of the filters to ensure accurate identification of individual PCs. This quantification is restricted to defined regions of interest and not the whole femur. However, the experimenter should keep consistency in imaging different bones for different conditions to be compared. The mouse model used in this study expresses RFP specifically in PCs following tamoxifen induction. Because this reporter line is not widely available, users working with other mouse models or non-reporter systems can adapt the workflow by substituting the RFP channel with any alternative plasma-cell-specific marker detectable by fluorescence imaging (e.g., CD138) conjugated to Alexa-555, Alexa 546, PE or similar fluorochromes.

## Troubleshooting

### Problem 1: Uneven trimming of the OCT block

If the femur is not embedded flat and properly aligned within the OCT block (Step 7), trimming may fail to remove the bone matrix uniformly along the diaphysis, resulting in incomplete BM exposure.

### Potential solution

Adjust the cryostat cutting angle slightly up or down to correct the trimming plane and avoid re-embedding. Alternatively, dissolve the OCT by immersing the block in 1X PBS until fully cleared, then re-embed the bone as described in the step-by-step method details section.

### Problem 2: OCT block breaking

Excessive force during trimming may cause the OCT block and the embedded bone to crack or break (Step 11).

### Potential solution

Adjust the advance of the OCT block to prevent it from hitting the blade forcefully during initial trimming. Trim gradually in thin sections to prevent mechanical fracture of the bloc.

### Problem 3: Loss of blue channel intensity at higher z-depth

**Figure 19:**
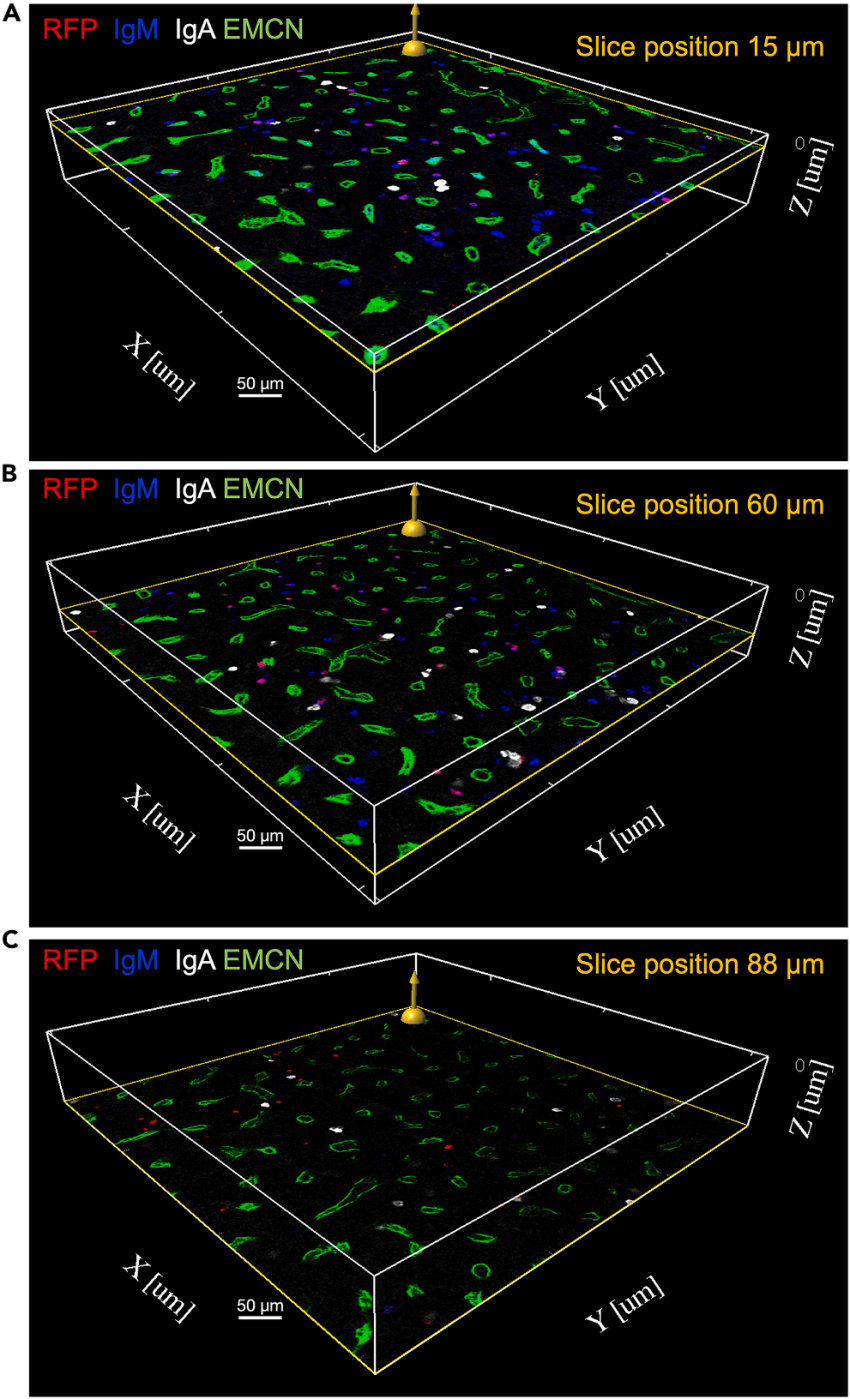
Orthogonal slice views of ROI 1, showing increasing z-depths from the surface The highlighted yellow plane indicates the slice position in the XY plane: (A) Slice position = 15 μm, (B) Slice position = 60 μm, (C) Slice position = 88 μm. IgM (Alexa Fluor 405, blue), IgA (Alexa Fluor 647, white), RFP (Alexa Fluor 555, red), Endomucin/EMCN (Alexa Fluor 488, green).

Longer-wavelength fluorophores (green, red, and far-red) are clearly visible at 88 μm, as illustrated in Figure 19. In contrast, the blue channel (Alexa Fluor 405) shows signal attenuation beyond ∼60 μm (Figures 19B and 19C), consistent with increased scattering and absorption of shorter wavelengths in thick tissues. These observations indicate depth-dependent attenuation of the blue signal, rather than limited antibody penetration.

### Potential solution

The user may omit the endomucin staining (Green) and use this channel for IgM detection at higher z-depth. For that, use a Goat anti-mouse IgM mu chain (Alexa Fluor 488 (Cat#ab150121) as an alternative to Goat anti-mouse IgM μ chain (Alexa Fluor 405 (Cat#ab175662).

### Problem 4: Autofluorescence of megakaryocytes

**Figure 20:**
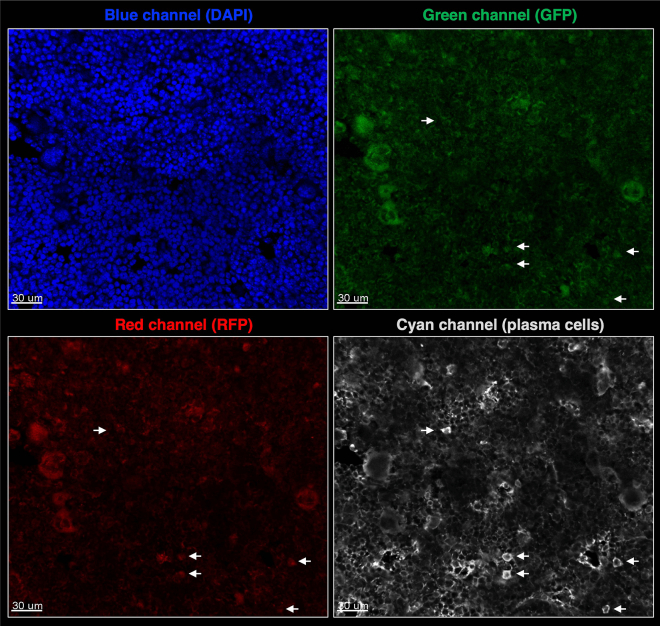
Representative 2D tile scan image of a femur showing natural autofluorescence of megakaryocytes Arrows: some plasma cells (pan Ig^+^ cells); Big cells: megakaryocytes.

Fluorescence from endogenously expressed fluorescent proteins (e.g., RFP, GFP) in our model was not detectable, or only weakly so, in fixed bone due to the strong megakaryocyte autofluorescence across multiple channels (see the green and red channels in Figure 20).

A femur from this mouse model was stained with Goat anti-mouse IgG, IgM, IgA (H+L) cross-adsorbed secondary antibody conjugated to biotin (Cat# A-10676, 1:500), revealed with Streptavidin Alexa Fluor 647 (Cat# S32357, 1:500), and counterstained with DAPI (1:1000 in 1× 1X PBS). However, endogenous GFP and RFP expressed in recombined plasma cells were not amplified. The representative image (Figure 20) shows individual channels: DAPI (blue), GFP (green), RFP (red), and plasma cell staining detected with Streptavidin Alexa Fluor 647 (cyan/gray). Megakaryocytes exhibit strong intrinsic autofluorescence, particularly in the green and red channels.

### Potential solution

When working with reporter mouse models (e.g., GFP, RFP, or mCherry), endogenously expressed fluorescent proteins must be amplified using an antibody-based detection strategy (primary antibody + secondary antibody, or biotinylated primary antibody + streptavidin conjugate) to ensure that reporter signals are detectable above the autofluorescence threshold of megakaryocytes.

### Problem 5: Red bone marrow after clearing

Bone remaining red after RapiClear treatment (steps 18-20) is highly unlikely. Persistent redness indicates incomplete removal of haemoglobin.

### Potential solution

• Confirm that fixation with 2% formaldehyde (Step 4) was carried out correctly, as excessive or prolonged fixation may reduce clearing efficiency.
• Ensure the bone was fully immersed in RapiClear while rotating.

## Resource availability

### Lead contact

Further information and requests for resources and reagents should be directed to and will be fulfilled by the lead contact, Dinis Pedro Calado (dinis.calado@crick.ac.uk).

### Technical contact

Technical questions on executing this protocol should be directed to and will be answered by the technical contact, Abdouramane Camara (abdou.camara@crick.ac.uk).

### Materials availability

This study did not generate new unique reagents.

### Data and code availability

This protocol did not generate new code.

## Acknowledgments

This work was supported by the 10.13039/100010438FCI, which receives core funding from 10.13039/501100000289Cancer Research UK (grant CC2078); the 10.13039/501100000265UK Medical Research Council (grant CC2078); the 10.13039/100010269Wellcome Trust (grant CC2078) to D.P.C.; the 10.13039/501100000265UK Medical Research Council (grant MR/W025221/1) to D.P.C.; the 10.13039/501100000377Vivensa Foundation (project grant AIS2110∖9) to D.P.C., A.Q.X., and A.C.; 10.13039/501100000268BBSRC Institute strategic programme grants BBS/E/B/000C0427 and BBS/E/B/000C0428 and 10.13039/501100000268BBSRC (grant BB/W016427/1) to D.P.C.; the European Molecular Biology Organization Postdoctoral Fellowship (grant EMBO ALTF 836-2021) to A.C.; and the Crick Africa Network (CAN) Career Acceleration Fellowship (grant PRJ_2076) to A.C. We thank the members of the Immunity and Cancer laboratory (FCI, London, UK) for critical discussions and comments. We thank the FCI scientific platforms (Biological Resource Facility and Advanced Light Microscopy) for expert advice and technical support. The schematics were created with BioRender.com.

## Author contributions

A.C. conceived the protocol. A.C. and D.P.C. acquired the funding. D.P.C. supervised the protocol. All authors participated in the writing of the manuscript.

## Declaration of interests

D.P.C. is a named inventor on a patent relating to the synthetic lethality of NMT inhibitors in high-MYC cancers (WO2020128475); D.P.C. is named inventor on a patent relating to Follicular Lymphoma biomarker signature (GB2509744.5). D.P.C. recieved research funding from AstraZeneca and Boehringer *Ingelheim*. These competing interests are unrelated to this work.
